# Decoding the Real-Time Neurobiological Properties of Incremental Semantic Interpretation

**DOI:** 10.1093/cercor/bhaa222

**Published:** 2020-08-31

**Authors:** Hun S Choi, William D Marslen-Wilson, Bingjiang Lyu, Billi Randall, Lorraine K Tyler

**Affiliations:** Centre for Speech, Language and the Brain, Department of Psychology, University of Cambridge, Cambridge CB3 0DX, UK; Centre for Speech, Language and the Brain, Department of Psychology, University of Cambridge, Cambridge CB3 0DX, UK; Centre for Speech, Language and the Brain, Department of Psychology, University of Cambridge, Cambridge CB3 0DX, UK; Centre for Speech, Language and the Brain, Department of Psychology, University of Cambridge, Cambridge CB3 0DX, UK; Centre for Speech, Language and the Brain, Department of Psychology, University of Cambridge, Cambridge CB3 0DX, UK

**Keywords:** Bayesian language modeling, electro/magnetoencephalography, incremental prediction, representational similarity analysis, semantics

## Abstract

Communication through spoken language is a central human capacity, involving a wide range of complex computations that incrementally interpret each word into meaningful sentences. However, surprisingly little is known about the spatiotemporal properties of the complex neurobiological systems that support these dynamic predictive and integrative computations. Here, we focus on prediction, a core incremental processing operation guiding the interpretation of each upcoming word with respect to its preceding context. To investigate the neurobiological basis of how semantic constraints change and evolve as each word in a sentence accumulates over time, in a spoken sentence comprehension study, we analyzed the multivariate patterns of neural activity recorded by source-localized electro/magnetoencephalography (EMEG), using computational models capturing semantic constraints derived from the prior context on each upcoming word. Our results provide insights into predictive operations subserved by different regions within a bi-hemispheric system, which over time generate, refine, and evaluate constraints on each word as it is heard.

## Introduction

Spoken language comprehension involves a variety of rapid computations that transform the auditory input into a meaningful interpretation. When listening to speech, our primary percept is not of the acoustic-phonetic detail, but of the speaker’s intended meaning. This effortless transition occurs on millisecond timescales, with remarkable speed and accuracy and without any awareness of the complex computations on which it depends. How is this achieved? What are the processes and representations that support the transition from sound to meaning, and what are the neurobiological systems in which they are instantiated?

Understanding the meaning of spoken language requires listeners to access the meaning of each word that they hear and integrate it into the ongoing semantic representation in order to incrementally construct a syntactically licensed semantic representation of the sentence (Tyler and Marslen-Wilson 1977; Marslen-Wilson and Tyler 1980; Kamide et al. 2003; Hagoort et al. 2009). Research to date provides a broad outline of the neurobiological language system and of the variables involved in language comprehension (Hickok and Poeppel 2007; Marslen-Wilson and Tyler 2007; Friederici 2011; Kutas and Federmeier 2011; Price 2012; Bornkessel-Schlesewsky and Schlesewsky 2013; Hagoort 2013; Matchin and Hickok 2020), but surprisingly, little is known about the specific spatio-temporal patterning and the neurocomputational properties of the incremental processing operations that underpin the dynamic transitions from the speech input to the meaningful interpretation of an utterance.

This is our goal in the present study where we probe directly the dynamic patterns of time-sensitive neural activity that are elicited by spoken words, focusing on the semantic constraints they generate on upcoming words and the incremental processes that combine them into semantically coherent utterance interpretations. We use computational linguistic analyses of language corpora to build quantifiable models of semantic constraint and mismatch, where the latter reflects the processing demands of interpreting the upcoming word given the properties of prior constraints (Hale 2001; Levy 2008). Based on these cognitive models, we employ representational similarity analysis (RSA) to probe the different types of neural computation that support dynamic processes of incremental interpretation, using source-localized MEG + EEG (EMEG) imaging to capture the real-time electrophysiological activity of the brain. RSA enables us to compare the (dis)similarity structure of our theoretically relevant models with the (dis)similarity structure of observed patterns of brain activity, revealing how different information types are encoded in different brain areas over time.

In a previous EMEG study, involving single spoken words, we used these methods to map out the spatio-temporal dynamics of the word recognition process (Kocagoncu et al. 2017). Using RSA to test quantifiable cognitive models of key analysis processes as they occur in real time in the brain, we identified the cortical regions that support the early phonological and semantic competition between cohort candidates as a word is heard, and the dynamic process of convergence on a single candidate and its unique semantic representation as the uniqueness-point (UP) approaches [i.e., the point at which the word can be differentiated from its word-initial cohort and is uniquely recognizable (Marslen-Wilson 1987)]. Hence, identifying the UP plays an important role in interpreting the timing of linguistic processing with respect to the input word. In a subsequent study, placing spoken words in a minimal phrasal context (e.g., yellow banana), we constructed RSA models of the semantic constraints generated by the adjective (yellow) to determine how these interacted with the processing of the following noun (banana). Consistent with previous behavioral and ERP results (Marslen-Wilson 1975; Kamide et al. 2003; DeLong et al. 2005; Bicknell et al. 2010), we found early effects of prior probabilistic semantic constraints on lexical processing (within 150–200 ms of word onset), where the timing of these effects reflects the prior access of potential word candidates driven by the sensory input (Klimovich-Gray et al. 2019). These studies suggest an underpinning lexical access process where lexical contents can be made available very soon after word onset for interaction with contextual constraints.

In the context of these two studies, the current study aims to determine how these rich contextual constraints incrementally combine words into a meaning interpretation and how this interpretation modulates the processing of subsequent words in the utterance. Critical to this study is the development of the appropriate quantifiable measures of the relevant properties of the sentential processing environment, as the basis for the RSA models used to probe the real-time brain activity elicited by hearing the test sentences.

Within the broad context of predictive processing frameworks (Kuperberg and Jaeger 2016), we investigated the role of semantic constraint elicited by the incrementally developing context in sentences such as “The experienced walker chose the path,” including its subject, verb, and object, in generating a message-level interpretation. To do this, we used language models of constraint and mismatch derived by combining the behavioral responses from sentence completion studies with the latent Dirichlet allocation (LDA) approach of topic modeling (Griffiths and Steyvers 2004). These models were used to construct RSA models of semantic constraints, as they evolve over a spoken utterance, and to look at the spatiotemporal pattern of model fit for each processing dimension being tested (Kocagoncu et al. 2017). Importantly, the cognitive models that test for effects of semantic constraints and their integration into the developing sentence are probabilistic and experiential in nature, reflecting language as people experience it in the real world and providing the type of quantifiable data necessary to calculate rich multivariate representational models. This avoids the limitation of relying on categorical distinctions between stimuli which fail to capture the multifaceted richness of linguistic representations and the probabilistic nature of language.

Our primary interest here is in what we call “combined constraints” on upcoming words, the cumulative constraints generated by the set of words comprising the prior context. In this study, we developed a set of contextual constraint models in order to illuminate the temporal progression of predictive processing as each word [i.e., verb and complement noun (CN)] incrementally unfolds over time. This enables us to illustrate the spatiotemporal dynamics of the cumulative effects of constraints and to determine how far these constraints are neurally expressed.

In common with recent accounts of incremental processing of speech inputs, we expect to see the computation of constraints as each word is being recognized (Marslen-Wilson 1975; Marslen-Wilson and Tyler 1980; DeLong et al. 2014). The RSA models, as described above, primarily focus on modeling these constraints and the relative timing with which they appear as the utterance unfolds over time. We also investigate the mismatch effect between the context and a target word (CN) that captures the difficulty of semantically processing the target word with respect to the constraint imposed by the prior context, based on its semantic properties. Together the timing and location of the effects captured by these models reveal a picture of when and where the human brain activates and utilizes constraints at the semantic level.

## Overview

To determine the spatiotemporal neural properties of incremental semantic interpretation during language comprehension, we developed models of the incremental constraints that the context imposes on the meanings of upcoming words and the mismatch between an upcoming word and its fit into the prior context. We tested these models against the spatiotemporal properties of the source-localized EMEG data to compare the similarity structure of our theoretically relevant models. We tested for the timing of the model fit generated for these models at different time points within a language mask that includes a set of brain regions comprising a bilateral fronto-temporo-parietal language system, which has been frequently reported in the literature (Binder et al. 2009). We asked when and where each of our key models—of semantic constraint, and mismatch—would fit the brain data, when and where is there an effect of the subject noun phrase (SNP) semantic constraint? how does it change as a subsequent verb is processed? and what is the scope of these constraint effects on upcoming words?

In order to model incrementally developing constraint over time, we obtained measures of semantic prediction at two different points in a sentence—immediately after the SNP [“the experienced walker”] and after the combination of the SNP + verb [“the experienced walker chose…”]. In this way, we aimed to characterize the changing patterns of prediction as a verb is combined with the initial SNP context. To do this, we conducted two separate behavioral studies with different participants in which they were asked to complete a sentence either after hearing the SNP fragments (study 1) or after hearing the SNP + verb fragments (study 2). We then extracted main verbs from the first behavioral study and CNs from the second behavioral study, allowing us to infer the predictive state of the brain throughout the sentence.

**Figure 1:**
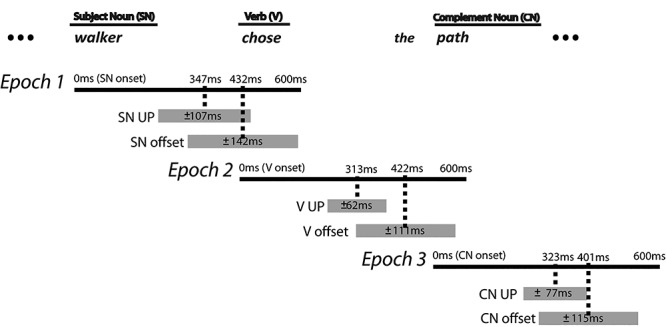
Overview of the epochs in the experiment in relation to the incremental processing: Epoch 1: Activation of SNP constraint; Epoch 2: Modification of SNP constraint based on the Verb; and Epoch 3: Evaluation of SNP + V constraint on CNs. The epochs were each defined relative to an alignment point (AP) such that Epoch 1 is aligned to the SN onset, Epoch 2 is aligned to the verb onset, and Epoch 3 is aligned to the CN onset. Each epoch lasted for 600 ms which included the average duration of each content word plus 1 SD. UP = the uniqueness point of a word (the earliest point in time when the word can be fully recognized after removing all of its phonological competitors).

However, in natural speech comprehension, prior constraints are relatively broad, so that specific words are rarely strongly predicted (Luke and Christianson 2016). Particularly, during the early stage of sentence processing, the context (SNP or SNP + verb) rarely provides a strong prediction of a particular upcoming word, leading to high uncertainty (entropy) in word-level constraints (Kuperberg 2016). Therefore, we applied topic modeling to each unique word provided by participants in the behavioral studies, in order to characterize constraints derived from the rich semantic (topic) representation associated with each unique word in a Bayesian framework of incremental predictive processing. To model prediction at a more abstracted semantic level, we combined the topic distributions of the continuation data into semantic “blends” of word candidates, modeling the conditional probability distribution $P\big({topic}/{full}\ {context}\big)$ (see Materials and Methods: IncrementalModels of Predictive Processing). Then, we computed entropy (see Materials and Methods: Spatiotemporal Searchlight RSA) of the blend to quantify the overall constraint strength, which was tested against the EMEG data during relevant epochs as described in Table 1 (see also Fig. 1), in order to investigate the incremental development of semantic constraint. Finally, in order to investigate how the constrained words are evaluated and incorporated into the prior context (SNP + verb), we also characterized the EMEG data using a pattern of mismatch between the predicted and the target semantics (see Materials and Methods: EMEG Recordings and MRI Acquisition).

**Table 1:** All semantic models used in this study and the epochs in which they were tested against the brain data. The epoch(s) in which each model was tested was chosen specifically to investigate the cascade of incremental predictive processes: 1) emerging with the early activation of the SNP constraint on verbs and on CNs before the verb is recognized; 2) evolving with a verb being incorporated into the context once the verb is recognized; and 3) facilitating the semantic interpretation once the CN is recognized. The average duration of each word to which each epoch is aligned is indicated by the bracket [mean ± standard deviation (SD)]

	Epochs (0–600 ms in duration)		
	Epoch 1: SN onset (432 ± 142 ms)	Epoch 2: verb onset (422 ± 111 ms)	Epoch 3: CN onset (401 ± 115 ms)
**Entropy**	SNP- > verbs SNP- > CNs	SNP- > verbs SNP- > CNs SNP + verb- > CNs	SNP + verb- > CNs
**Mismatch**	−	−	SNP + verb<-CN

In light of the claims that semantics is represented bilaterally (Price 2010, 2012; Wright et al. 2012), our approach provides an opportunity to determine whether different kinds of semantic computations are represented differentially across the hemispheres. We expected the predictive computations based on this information to involve bilateral anterior temporal and frontal areas with the right hemisphere (RH) involved in the construction of a broader semantic representation and the engagement of the context (Beeman and Chiarello 1998; St George et al. 1999; Seger et al. 2000; Jung-Beeman 2005).

## Materials and Methods

### Participants

Fifteen participants (7 females; average age: 24 years; range: 18–35 years) took part in the study. They were all native British English speakers and right-handed with normal hearing. Two participants were excluded from the analysis: one because of sleepiness during the EMEG study and the other because of poor quality EEG recordings. Informed consent was obtained from all participants and the study was approved by the Cambridge Psychology Research Ethics Committee.

### Stimuli

We constructed 200 spoken sentences consisting of an SNP (e.g., “the experienced walker”), followed by a verb (e.g., “chose”) which in turn was followed by a CN (e.g., “path”). The sentence sets were constructed in the following way. First, we chose verbs from the VALEX database (Korhonen et al. 2006) that occurred with (at least) two different complement structures: one was a simple transitive direct object (DO) structure (e.g., “… chose the path…”) and the other was one of three other possible complement structures including sentential complement (SC; “… denied that the court …), infinitival complement (INF; “… wanted to become *…*”), and prepositional phrase complement (PP; “… fled to the forest …”). For 72% of the stimuli, the DO complement structure was more frequent [according to the subcategorization frame (SCF) information in VALEX; (Korhonen et al. 2006)] with the average probability of 0.499 ± 0.12 (mean ± SD). By adding some variability to the function words of the complement phrase, we aimed to improve the generalizability of our results to any natural spoken sentence with varying subcategorization structures.

**Figure 2:**
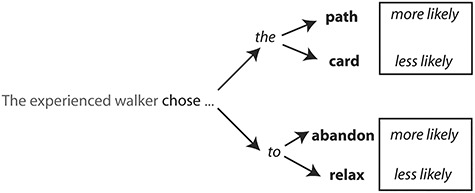
Design of the experimental stimuli. Each sentence contained a key main verb (“chose”) followed by a complement function word (“the” or “to”) to vary the complement in terms of the SCF preference of a preceding verb. A function word was followed by a noun or a verb that was either consistent with the verb’s preferred continuation or less preferred continuation.

To ensure variability in the predictability of the CNs, we varied the probability of these nouns with the preceding verb and the complement function word according to Google Books n-gram frequencies. Note that this variability was controlled when running the analysis by including the frequency of a word to which the epoch was aligned to as one of the covariates and partialling out when correlating the data and model representational dissimilarity matrices (RDMs) [e.g., SN frequency at epoch 1, verb frequency at epoch 2, and CN (content word) frequency at epoch 3]. This process resulted in 200 sentences with four repetitions of the SNP + verb combination (see Fig. 2), consisting of varying complement structures (i.e., DO, SC, INF, and PP) with different complement content words. This ensured sufficient variability between trials in the ease with which the content word in the complement could be integrated into the ongoing sentential representation, given the constraints provided by the preceding context. Just as for the lexical frequency, we controlled for the repetition effect of the SNP + verb combination by including it as another covariate. In summary, we partialled out the effects of 1) lexical frequency of a word to which an epoch is aligned and 2) repetition of stimuli across trials.

The sentences were spoken by a native female British English speaker and were recorded in a soundproof booth. In the experiment, participants were asked to listen to these sentences attentively while we recorded their brain activity using EMEG. There was no explicit task for them to perform since tasks are known to invoke domain general brain systems over and above any domain-specific language effects (Campbell and Tyler 2018). All stimuli were pseudo-randomized and counter-balanced across participants. We followed the standard procedure for presenting auditory stimuli as in our previous studies (Kocagoncu et al. 2017; Klimovich-Gray et al. 2019).

### Incremental Models of Predictive Processing

In this study, we focused on the two different incremental computations: 1) constraint and 2) evaluation in order to investigate the neurobiological underpinnings of how the preceding context guides the interpretation of an upcoming word. To do this, we combined behavioral data with computational models of semantics as described below.

#### Behavioral Studies

To model incrementally evolving constraints over the SNP, verb, and CN, we conducted two separate behavioral studies. In the first experiment, 24 participants (who did not take part in the main experiment or the second behavioral study) heard each unique SNP (e.g., “The experienced walker …”) and provided a sentence continuation after the SNP (e.g., “… hiked through the mountains,” “… chose a less travelled path,” etc.). We extracted the main verb from each sentence continuation and used these data with topic representations (see Materials and Methods: Stimuli) to capture predicted verb semantics. In the second experiment, we asked 31 participants (who did not take part in the main experiment or the first behavioral study) to provide a sentence continuation after hearing each unique SNP + verb in our stimuli (e.g., “The experienced walker chose …”), for example, “… the shorter route,” “… the hardest path,” etc. Note that we only used the noun responses which are considered to be an object of the preceding verb (e.g., nouns in DO or PP complements which we refer to as CNs throughout this paper) in order to remove any syntactic or thematic variability when modeling semantic interpretation of the CN. For example, any noun responses in an SC were removed since they are often treated as a new subject instead of an object (e.g., “The walking couple heard that the **farm** was open to visitors”). On average, this left 18 CN responses for every stimulus from 31 participants. Any stimulus with less than 4 responses were excluded from the analysis.

#### Semantic Modeling

We trained a probabilistic topic model based on LDA (Griffiths and Steyvers 2004). It develops a generative probabilistic model that assigns a word to different latent dimensions in a way that maximizes the posterior of the model. Such latent dimensions are often called “topics” which describe the semantic content of a word in the form of a probability distribution. In this study, topic distributions (consisting of 100 topics) associated with each content word were generated using corpus-based tensor data (Baroni and Lenci 2010). Instead of using raw co-occurrence frequency, we used local mutual information from the tensor because it normalizes the effect of lexical frequency of individual items when computing the semantic relation (co-occurrence) between two words. Furthermore, instead of using all co-occurrence data in the tensor, we only selected specific subsets in order to capture syntactically licensed semantic representation specifically with respect to a word in the context. In particular, we focused on the incremental and cumulative development of the semantic constraint from an SN (agent) to a CN. To do this, we trained two separate topic models based on the co-occurrence between 1) SN and verb (SN-V) and 2) the preceding words including SN and verb and CN (object) (SNV-CN). These models provided different aspects of semantic representation relevant for incremental predictive processing as follows: 1) the first (SN-V) topic model was trained specifically to characterize the predictive representation of SNs on upcoming verbs and the specific semantic content of verbs that are syntactically licensed with respect to the preceding SNs and 2) the second (SNV-CN) topic model was trained specifically to characterize the predictive representation of SNs and verbs on CNs and the specific semantic content of CNs that is syntactically licensed with respect to the preceding SNs and verbs. See Section 1 in Supplementary Materials for more details regarding model training and parameter settings. See Supplementary Fig. S1 for illustrations of SNV-CN topic model.

#### Modeling Predictive State: Semantic Blends

After obtaining the behavioral responses from the two sentence completion studies (verbs from the first and CNs from the second study) and the topic representation associated with a set of unique responses for each sentence, we combined them to generate an overall representation across multiple responses (for either the unique verbs or the CNs) to capture consistent semantic content shared by the set of verbs predicted by the SNP or by the CNs predicted by the SNP + verb. In this way, we aimed to model predictive activation of semantic contents associated with multiple lexical items based on the preceding context. The semantic blend was computed as below

$$\begin{eqnarray*}&& \mathrm{blend}\left(\mathrm{word}\mathrm{s}\right)=P\left(\mathrm{topic}|\mathrm{full}\ \mathrm{context}\right)\\ && \qquad=\sum_{\mathrm{word}}P\left(\mathrm{topic}|\mathrm{word}\right)P\left(\mathrm{word}|\mathrm{full}\ \mathrm{context}\right), \end{eqnarray*}$$

where $P\big(\mathrm{word}|\mathrm{full}\ \mathrm{context}\big)$ is a probabilistic weight associated with a given $\mathrm{wor}$d (see Behavioral Studies) and $P\big(\mathrm{topic}|\mathrm{word}\big)$ is the topic distribution for $\mathrm{word}$ (see Incremental Models ofPredictive Processing). Based on this formula, we constructed three different “blend” vectors.

##### SN-V verb blend

This blend is designed to model the SNP constraint on upcoming verbs. We counted the (post-SNP) verb responses from the first sentence completion study. Then, the frequency count associated with each unique verb that was produced by participants was, in turn, used as a weight to the topic distribution of the verb. From the topic model trained specifically on the SN-verb co-occurrence data, we obtained the topic representation of each unique verb which was weight-combined as expressed in the formula above (i.e., $P\big({verb}\_{topic}|{verb}\big)P\big({verb} / {SNP}\big)$).

##### SNV-CN verb blend

Despite being a verb blend, this second blend model is designed to model the SNP constraint on CNs (rather than its constraints on the verb), via the set of predicted verbs obtained from the first behavioral study. We counted the (post-SNP) verb responses and the frequency count associated with each unique verb that participants produced as above. However, we obtained the verb topic distributions from a second topic model trained specifically on the mixed SN-CN and verb-CN co-occurrence data, reflecting the predictive representation on upcoming CNs. Then, each predictive representation (topic-context distribution) of unique verbs in relation to CNs was weight-combined as expressed in the formula above (i.e., $P\big(\mathrm{CN}\_\mathrm{topic}|\mathrm{verb}\big)P\big(\mathrm{verb}|\mathrm{SNP}\big)$).

##### SNV-CN CN blend

The third blend focused on modeling the combined constraint of SNP + verb on CNs. To do this, we counted the (post-SNP + verb) CN responses from the second sentence completion study. Then, we used the CN topic distributions from the second topic model trained specifically on the mixed SN-CN and verb-CN co-occurrence data, reflecting the topic representation of each unique CN in relation to the preceding subjects and verbs. Then, just as the other blends, each topic representation (target-topic distribution) associated with each unique CN was weight-combined as expressed in the formula above (i.e., $P\big(\mathrm{CN}\_\mathrm{topic}|\mathrm{CN}\big)P\big(\mathrm{CN}|\mathrm{SNP}+\mathrm{verb}\big)$).

In summary, we generated the following blends whose entropy is designed to address how constraints incrementally change and develop.

1. $P\big(\mathrm{verb}\_\mathrm{topic}|\mathrm{SNP}\big)=\sum_{\mathrm{verb}}P\big(\mathrm{verb}\_\mathrm{topic}|\mathrm{verb}\big)P\big(\mathrm{verb}|\mathrm{SNP}\big)$.
2. $P\big(\mathrm{CN}\_\mathrm{topic}|\mathrm{SNP}\big)=\sum_{\mathrm{verb}}P\big(\mathrm{CN}\_\mathrm{topic}|\mathrm{verb}\big)P\big(\mathrm{verb}|\mathrm{SNP}\big)$.
3. $P\big(\mathrm{CN}\_\mathrm{topic}|\mathrm{SNP}+\mathrm{verb}\big)=\sum_{\mathrm{CN}}P\big(\mathrm{CN}\_\mathrm{topic}|\mathrm{CN}\big)P\big(\mathrm{CN}|\mathrm{SNP}+\mathrm{verb}\big)$.

#### Modeling Predictive Constraint: Entropy

Entropy is a metric designed to quantify the amount of uncertainty in distributional models. Therefore, entropy of the blend distributions in this study reflects the strength of semantic constraint regarding upcoming words (higher uncertainty = weaker constraint). However, in any topic models, each topic varies in terms of the types of words it prefers with different probabilities. This naturally leads to variations in semantic dispersion across topics, potentially undermining the estimation of true semantic entropy. Here, we addressed this issue by linearly combining entropy with topic dispersion as following:

$$ H\left(P(x)\right)=w\ast h\left(P(x)\right)=\sum_i{w}_i\left[-P\left({x}_i\right)\log P\left({x}_i\right)\ \right], $$

where $w$ is a vector of semantic dispersion across topics and $h\big(P(x)\big)$ is a vector containing local entropy values. In this paper, we denote the term entropy and notation $H$ to refer to this dispersion-corrected entropy. The semantic dispersion was calculated by averaging pair-wise cosine distances between topic distributions among every pair of words within a topic (Lyu et al. 2019). If the target words preferred by a topic have similar distributions, the average cosine distance will be low. Then, this “within-topic” semantic dispersion was linearly combined with the local entropy values to manipulate the contribution of each topic to the degree of overall constraint strength across topic candidates. In this way, we effectively controlled for “within-topic” dispersion when computing “between-topic” constraint.

**Figure 3:**
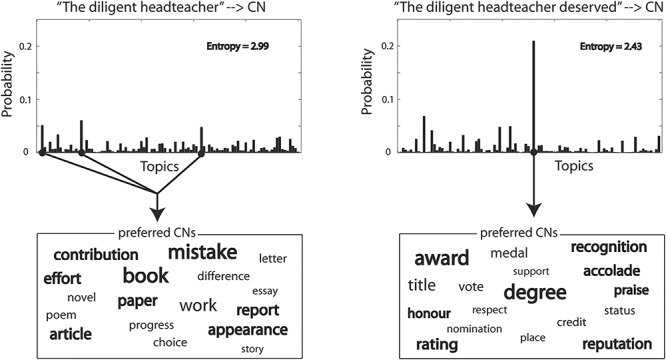
Reducing entropy in prediction before (left panel) and after (right panel) a verb is incorporated into the SNP context. The topic distributions on the top are the semantic blends of predicted CNs by SNP and SNP + verb, respectively. Entropy associated with each of the two distributions is also described. The word boxes below the distributions show a set of preferred words based on the predicted topics.

Each of the semantic blends described above was taken as an input to the entropy function (Fig. 3), generating three semantic constraint models which were tested against the spatiotemporal patterns of neural activity at specific epochs (Table 1).

1. SNP adjacent constraint model on upcoming verbs: entropy of $P\big(\mathrm{verb}\_\mathrm{topic}|\mathrm{SNP}\big)$.
2. SNP nonadjacent constraint model on upcoming CNs: entropy of $P\big(\mathrm{CN}\_\mathrm{topic}|\mathrm{SNP}\big)$.
3. SNP + verb constraint model on upcoming CNs: entropy of $P\big(\mathrm{CN}\_\mathrm{topic}|\mathrm{SNP}+\mathrm{verb}\big)$.

#### Modeling Evaluation: Constraint Mismatch

Semantic evaluation refers to a process of resolving mismatch between a current input and the predicted candidates based on the preceding context, leading to an accurate interpretation of the input that fits the context. To model this process, we quantified the degree of mismatch by computing cosine distance between the semantic representations of the predicted CNs and the target CN. As described in Materials and Methods: Behavioural studies, we excluded any items that do not contain CN (i.e., a noun considered to be an object of a preceding verb) from the analysis because this mismatch model requires the target CN to be identified. This left us with 128 out of 200 trials.

### Spatiotemporal Searchlight RSA

**Figure 4:**
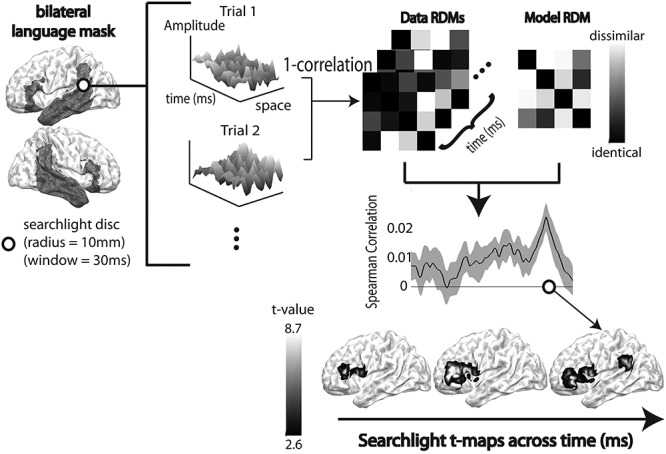
A schematic illustration of the searchlight RSA of spatiotemporal source-space EMEG data. The bilateral language mask used in this study is surface-rendered onto the brain template in the figure for visualization. Since the source-space EMEG data inherently vary across time and space, we calculated the similarity of the spatio-temporal patterns of brain activities for different trials based on measurements within each searchlight sphere with a spatial radius of 10 mm and a temporal radius of 30 ms. We used 1—Pearson’s correlation between pairs of trials as the distance metric to compute an RDM for each searchlight, yielding a searchlight map of data RDMs. Each data RDM is then correlated with each model RDM using Spearman’s correlation. This Spearman’s correlation was computed for each subject and the significance of the correlation at each searchlight location was tested using one-sample *t*-test (H0: Spearman correlation will be zero). The figure illustrates this process, yielding a time-course of *t*-values across spatiotemporal searchlights.

In order to determine when and where these constraint models and associated computations are neurally realized, we used spatiotemporal searchlight RSA (ssRSA) (Su et al. 2012). Each searchlight is defined for each vertex at each time-point, providing a fine-grained spatiotemporal map of neural activity. To characterize such dynamic pattern of neural activity, we constructed model RDMs using specific properties of the blended distributions across sentences described above. Since all of the model RDMs in this study were based on the summary metrics designed to capture various incremental aspects of distributional semantics, the representational geometry was characterized simply by calculating the absolute distance of the metric values between every pair of trials. Each of these model RDMs was, then, compared with the patterns expressed by the neural RDMs constructed by correlation distance between every pair of trials for each searchlight across space and time (see Fig. 4). The size of each searchlight was set as a spatial radius of 10 mm and a temporal radius of 30 ms.

ssRSA was performed within a language mask, which included all anatomical regions in a set of regions encompassing bilateral fronto-temporo-parietal regions, using the Harvard-Oxford cortical atlas (Kocagoncu et al. 2017; Lyu et al. 2019). See Figure 4 for surface rendering of this language mask. These regions are reliably shown to be involved in language processing (Binder et al. 2009; Price 2010, 2012).

### EMEG Recordings and MRI Acquisition

MEG data were recorded on a VectorView system (Elekta Neuromag) using 306 sensors (102 magnetometers and 204 planar gradiometers), located in a magnetically shielded room at the MRC Cognition and Brain Science Unit, Cambridge, UK. In conjunction with the MEG recordings, we recorded EEG signals using an MEG compatible EEG cap (Easycap, Falk Minow Services) with 70 electrodes, plus external electrodes and a nose reference. To monitor head movement in the MEG helmet, five head positioning indicator (HPI) coils attached to the scalp recorded head position every 200 ms. Blinks and eye movements were recorded by electro-oculogram (EOG) placed above and beneath the left eye and beside the left and right outer canthi. Cardio-vascular effects were recorded by electro-cardiogram (ECG) attached to right shoulder blade and left torso. To be able to co-register the EEG and MEG data to anatomical structural scans for each participant, the positions of the HPI coils and EEG electrodes were digitized relative to three anatomical landmarks (nasion, left and right peri-auricular points). In addition, a participant’s head shape was digitized across the head. MEG signals were recorded with a sampling rate of 1000 Hz and any signals below 0.03 Hz were high-pass filtered.

To localize the EEG and MEG data to sources on the cortical surface, structural MRI scans were acquired for each participant in a separate session using 1-mm isotropic resolution *T*_1_-weighted MPRAGE on a Siemens 3 T Prisma scanner (Siemens Medical Solutions) located at the Cognition and Brain Science Unit, Cambridge, UK.

### EMEG Preprocessing

The raw MEG data were max-filtered (Elekta-Neuromag) to remove bad channels, to compensate for head movement using signal space separation techniques (Taulu and Simola 2006).

Statistical parametric mapping 8 (SPM8; Welcome Institute of Imaging Neuroscience) was used to complete the remaining stages of EMEG preprocessing [except for independent component (IC) analysis artifact rejection]. First, a low-pass filter at 40 Hz was applied to the data using a fifth-order bidirectional Butterworth digital filter. In order to remove any physiologically driven artifacts such as blinks or cardiac signals recorded by EOG and ECG, the data signals were decomposed into ICs and each IC was correlated with vEOG, hEOG, and ECG channels. Any ICs showing very high temporal correlation (correlation >0.3) with any of these channels were removed and the remaining ICs were then visually inspected to ensure that no artifact component remained. The remaining ICs were then used to reconstruct the data.

Next, three separate analysis epochs were generated by aligning the data to the onset of each of the three points of interest in each sentence (see Fig. 1). The duration of each epoch (0–600 ms) was consistent across all three epochs. This duration was chosen to cover the average duration of each word + 1 SD described in Figure 1. One epoch was aligned to the SN, another to the verb, and a third to the CN. We also calculated the uniqueness point (UP) of each of these words from CELEX database (Baayen et al. 1993) to relate the timing of neural effects to when the word is recognized.

After epoching, each channel was baseline-corrected by subtracting the time-averaged data from a baseline period −200 to 0 ms relative to sentence onset (i.e., a period of silence immediately preceding the sentence). Finally, automatic artifact rejection was used to identify trials for which 15% or more sensors in any one of the three sensor types exceeded amplitude threshold (6e−11 T for magnetometers, 3e−12 T/m for gradiometers, and 2e−04 V for EEG), and these trials were rejected [an average of 15 trials were rejected (SD = 13.43)].

### EMEG Source Reconstruction

Source reconstruction aims to estimate the regional response within a brain using the EMEG data recorded outside the scalp. We first transformed the participants’ structural MRI images into an MNI template brain, which was then inverse-transformed to construct individual scalp and cortical meshes by warping canonical meshes of the MNI template brain to the original MRI space (Mattout et al. 2007). The MRI co-ordinates from individual scalp and cortical meshes were co-registered with the MEG sensor and EEG electrode co-ordinates by aligning fiducial points and the digitized head shape to the outer scalp mesh. A single-shell conductor model and a boundary element model were used as forward models for MEG and EEG recordings, respectively (the defaults in SPM8). We source-reconstructed our data based on the minimum-norm assumption in SPM8 as a prior on the source covariance (López et al. 2014). This source prior was empirically adapted to maximize the model evidence, which, in turn, was used to compute the Maximum A Posterior (MAP) source estimate.

### Statistics and Multiple Comparisons Correction

Using the correlation time-courses for the model and data RDMs across subjects, we calculated a time-course of one-tailed *t*-statistic for every vertex (Fig. 4). From this point-wise statistic, we applied the cluster forming threshold (CFT) of *P* = 0.01 and binarized the time-courses into clusters from a set of temporally and spatially contiguous vertices (data-points). Then, we summed *t*-values across each of the vertices within a cluster to compute a cluster-summed *t*-value. In this way, we aimed to emphasize the neural clusters that are spatiotemporally distributed, while each of the vertices in the clusters shows *P*-value less than 0.01.

For multiple comparisons correction across time-points which are not independent of one another, we ran permutation statistics (Maris and Oostenveld 2007) on the CFT output. Under the null hypothesis that our model is not correlated with the data (*r* = 0), we randomly permuted the sign of correlation values across different subjects and ran one-sample *t*-test for every time-point. For each randomization, this null time-course of *t*-values was converted to the time course of cluster-summed *t*-statistics. This random permutation process was repeated 1000 times and the cluster with the maximum *t*-value across all data-points for every run was saved. This process gives 1000 cluster-level *t*-values under the null hypothesis and the significance of the observed cluster-level *t*-values were evaluated with respect to this null distribution.

## Results

Using RSA and model RDMs of semantic constraint and mismatch, we probed source-localized EMEG data capturing the real-time electrophysiological activity of the brain to determine the spatiotemporal properties of the cumulative incremental effects of semantic constraints. For this purpose, we directly compared the strength of semantic constraints generated by the SNP on verbs and CNs, as quantified by the entropy of $P\big(\mathrm{verb}\_\mathrm{topic}|\mathrm{SNP}\big)$ and $P\big(\mathrm{CN}\_\mathrm{topic}|\mathrm{SNP}\big)$, against the multivariate patterns of neural activity over space and time. Then, we looked at the effects of the combined SNP + verb constraint by computing entropy of $P\big(\mathrm{CN}\_\mathrm{topic}|\mathrm{SNP}+\mathrm{verb}\big)$. In this way, we aimed to investigate the timing and neural regions that are related to generating semantic constraints prior to a target word (i.e., verb or CN). At last, to measure the predictive effects of the incrementally developed constraint on the processing of the CN semantics, we constructed a constraint mismatch model to examine the neural effects of semantic evaluation. We report significant (*P* ≤ 0.05) and marginally significant (0.05 < *P* ≤ 0.06) effects of the models sequentially as the sentence unfolds over time. Note that all of these reported results have large effect sizes (*d* > 0.8; See Fig. S2 in Supplementary Materials Section 2).

### SNP’s Adjacent Semantic Constraint (Entropy) on Upcoming Verb

**Figure 5:**
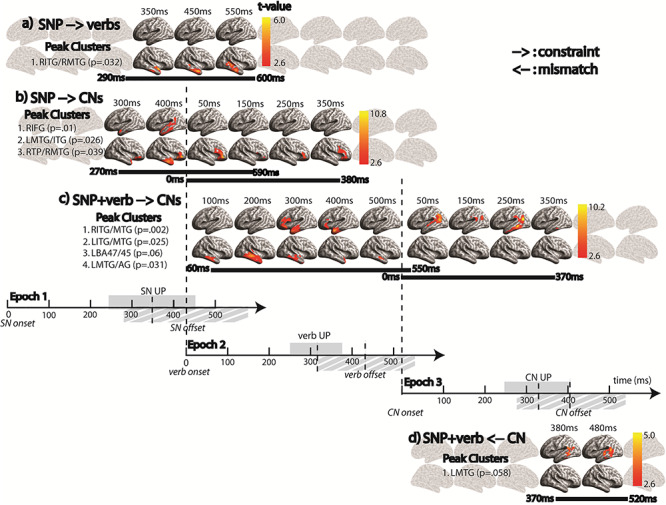
Results of the ssRSA with the constraint and mismatch models across three epochs described in Figure 1. Each panel shows the results for different models, corresponding to each subsection in the Results. All clusters were corrected by permutation statistics with the CFT of *P* = 0.01 and cluster-wise significance threshold of *P* = 0.05 (note that marginally significant clusters with *P*-values between 0.05 and 0.06 are also reported). A horizontal bar in black indicates the duration of the given cluster. The three alignment points [SN (subject noun), verb, and CN onsets] are indicated by long vertical dotted lines. UP stands for “uniqueness point” estimated by the CELEX database and the shaded region in gray around the mean UP reflects ±1 SD from the onset. Similarly, the mean offset of each word is also marked and the region shaded by gray hatch lines around the mean offset reflects ±1 SD from the onset.

We anticipated that the semantics of the SNP (e.g., “The experienced walker”) would generate rich constraints on the upcoming speech. To test this hypothesis, we constructed models capturing the strength of constraints generated by the SNP (e.g., entropy of $P\big(\mathrm{verb}\_\mathrm{topic}|\mathrm{SNP}\big)$ in this section and $P\big(\mathrm{CN}\_\mathrm{topic}|\mathrm{SNP}\big)$ in the section below). Using these entropy models, we aimed to assess the earliness of predictive computations and how they develop throughout a sentence. The results (Fig. 5*a*) show that the constraints on the verb generated by the SNP are significantly activated around the UP (347 ± 107 ms after the onset) of the SN as it is recognized, lasting around 300 ms from 290 to 600 ms, and are seen primarily in RH mid-anterior middle and inferior temporal areas (*P* = 0.032). This effect continued until the end of the SN (Epoch 1) and was not significant in Epoch 2, suggesting that listeners are actively constraining upcoming verbs as soon as they recognize the SNP and that these constraints involve only RH temporal regions.

### SNP’s Nonadjacent Semantic Constraint (Entropy) on CN

When examining constraints on nonadjacent words in a sentence (in this case, SNP constraints on the CN), we need to consider the semantic relation between the context (SNP) and the target (CN) while taking into account any words that intervene between them (in this case, the verb). Using the Bayesian approach, we computed the nonadjacent SNP constraint on CNs by taking into account the set of verbs that were predicted by hearing the SNP in the first behavioral completion study: $\sum_{\mathrm{verb}}P\big(\mathrm{CN}\_\mathrm{topic}|\mathrm{verb}\big)P\big(\mathrm{verb}|\mathrm{SNP}\big)$. This mathematical formulation reflects the SNP constraint on CN semantics via the set of verbs predicted by the SNP collected from the first behavioral study. This set of predicted verbs can be thought of as a process of semantic competition among partially activated semantic candidates. This is similar conceptually to the notion of cohort competition for spoken language comprehension [see (Marslen-Wilson 1987) which claims that multiple, partially activated word candidates initiated by the accumulating speech input as a word is heard momentarily compete with each other until the word is recognized]. Applying topic modeling to these predicted verbs enables us to model the SNP’s constraints on the CN taking into account the scope of the SNP’s prediction on the intervening verb.

Similar to the SNP’s constraint on verbs, this nonadjacent constraint appeared around the UP of the SN starting from 270 to 590 ms after the SN onset (Fig. 5*b*). It involved early, relatively short-lived effects in bilateral anterior and middle temporal cortex [left hemisphere (LH): *P* = 0.026 from 280 to 510 ms; RH: *P* = 0.039 from 280 to 530 ms], which overlapped with effects in right inferior frontal areas (*P* = 0.026 from 270 to 590 ms; see Fig. 5*b*). Note that these are the results from Epoch 1 aligned to the SN onset.

In a further analysis, we tested the spatiotemporal patterns of neural activity with the same nonadjacent SNP constraint model in Epoch 2 (Fig. 5*b*). We found a significant SNP semantic constraint effect on the CN but only in the right inferior frontal gyrus (RIFG) from the verb onset (*P* = 0.01; Fig. 5), lasting for 380 ms (1 SD after the mean UP), suggestive of competitive processing. We discuss the differential role of RIFG from the RH temporal regions in light of the constraints that they activate in the Discussion.

### SNP + Verb’s Semantic Constraint (Entropy) on CN

The analysis above examined the effect of the constraints imposed by the SNP on the CN mediated through verbs predicted in the behavioral test. In this section, we investigate the changes in the semantic constraint on CN as the SNP context becomes enriched by combining with its adjacent verb (i.e., after the cohort competition among the verb candidates has ceased, a process reflected in the blend model). To do this, we tested the effect of the SNP + verb constraint model on CNs [i.e., entropy of $P\big(\mathrm{CN}\_\mathrm{topic}|\mathrm{SNP}+\mathrm{verb}\big)$], in order to elucidate the neurobiological basis of the development of incremental constraints (Fig. 5*c*). Our results showed that right mid-anterior middle and inferior temporal areas again played a role in constraining the CNs from 60 ms after the verb onset and lasting around 500 ms (*P* = 0.002; Fig. 5*c*). This early constraint effect likely reflects the constraint driven by the event generated by the SNP, which could be largely consistent with the constraint imposed by the verb, especially when the verb is light in terms of its semantic constraint as in the majority of our sentence stimuli (see Discussion). In addition, we also found a significant cluster in left anterior middle and inferior temporal regions from 270 to 470 ms (*P* = 0.025) and a marginally significant cluster in left inferior frontal gyrus [LIFG (BA47/45); *P* = 0.06]. Based on the involvement of LATL and LBA47/45 in constraining upcoming CNs around the UP of the verb, we speculate that their role is to unify the verb into the broad semantic constraint setup by the SNP, essentially leading to a reduction in uncertainty in the constraint (see Fig. 3 and Fig. S3-1 in Supplementary Materials Section 3).

The effect of the combined SNP + verb constraint persisted into Epoch 3 during which the CN is heard. This lasted until 370 ms into the CN, which is around the UP. However, this transition was associated with more posterior LH regions in middle temporal gyrus (MTG) and angular gyrus (*P* = 0.031; Fig. 5*c*). This anterior-to-posterior transition may underscore the process from constructing to utilizing the context-driven semantic constraint when hearing the CN in a sentence.

### Semantic Mismatch between the Target CN and the Predicted CNs by the SNP + Verb Context

Our final analysis was aimed at demonstrating how the prior SNP + verb constraint facilitates the interpretation of the CN in light of its preceding context. To do this, we computed the cosine distance between topic representation of the target CN (i.e., $P\big(\mathrm{CN}\_\mathrm{topic}|\mathrm{CN}\big)$) and blend representation across the predicted CNs by the preceding SNP + verb context (i.e., $P\big(\mathrm{CN}\_\mathrm{topic}|\mathrm{SNP}+\mathrm{verb}\big)$). This model reflects the degree of mismatch between the predicted and the target semantics of the complement. This measure can be viewed as an index of semantic evaluation as it indicates the difficulty of processing the CN in light of the preceding context. Using this model, we observed a cluster marginally significant (*P* = 0.058) in LH posterior MTG from 370 to 520 ms after the CN onset (Fig. 5*d*). The timing of this mismatch effect emerges just after the constraint effect disappears, suggesting that the constraint is evaluated against the CN as soon as the predictive process terminates and the CN is fully identified. This last piece of evidence sheds light on the predictive computations actively engaged by listeners while incrementally processing the subject, verb, and object, which are critical components of understanding the message that speaker conveys.

## Discussion

The goal of the present study was to understand the neural dynamics of cognitive processes as listeners incrementally interpret the spoken sentences that they hear. The computations involved in this process include: 1) the activation of the semantic constraints generated by the semantic content of each word in a sentence as it is heard based on activated broad scenarios [or event structures]; 2) how and when these constraints affect processing of the upcoming speech; and 3) the incremental fine-tuning and evaluation of the semantic constraint on each new word, integrating it into the developing semantic representation. During the experiment, listeners heard sentences consisting of an SNP, followed by a verb, and then a CN where the SNP and the verb varied in the cumulative probabilistic constraints they generated on the upcoming complement. We tested for the timing and neural location of these computations by recording real-time brain activity using EMEG and analyzing the spatiotemporal fit of patterns of probabilistic topic models with source-localized neural activity across an extensive set of bilateral frontal, parietal, and temporal regions.

**Figure 6:**
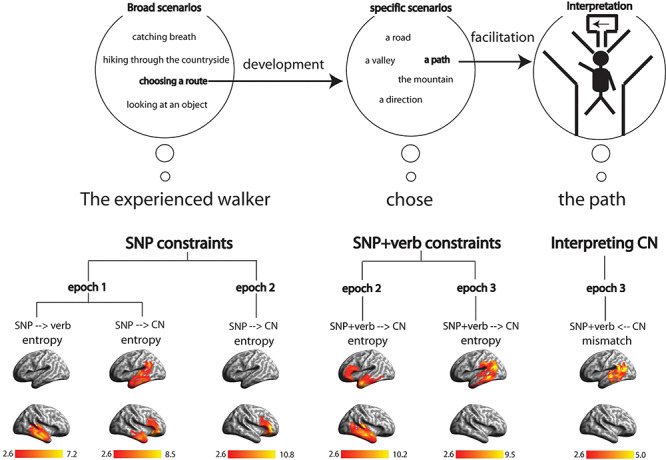
Vertex-wise peak *t*-value across three different epochs, summarizing the time-course of *t*-statistics. Above the surface rendering of each effect, cognitive implications of incremental constraints are illustrated: 1) activation of broad constraints primarily in right mid-anterior temporal lobe, which additionally engages left temporal and right inferior frontal regions possibly due to the grammatical category and adjacency of a word (CN) being constrained; 2) developing constraints recruits LIFG-LATL regions that reduce the amount of uncertainty (competition) in the activated constraints; and 3) as a constrained word (CN) is being heard, the specific constraint interacts with the bottom-up input, facilitating its processing in posterior middle temporal and inferior parietal areas.

Our summary of the results with respect to the timing of effects throughout the entire sentence reveals the rapid transitions of information processing in the brain as each word (SN, verb, and CN) incrementally unfolds over time. Such transitions highlight the underlying neural computations not only involved in processing individual words, but also in combining them with the prior context to develop a representation of the meaning of the sentence (see Fig. 3 and Fig. S3-1 in Supplementary Materials Section 3). More specifically, our results revealed the spatiotemporal dynamics of incremental semantic computations in the brain: 1) the early activation of semantic constraints generated by the SNP primarily engaged RH mid-anterior temporal areas whereas activating the non-adjacent constraint on CNs additionally recruited the RIFG and left temporal regions; 2) as the verb is recognized, the RH clusters started to decline but new clusters emerged in anterior left IFG (LIFG) and left anterior temporal lobe (LATL), actively constraining CNs based on the combined SNP + verb context; 3) as the target word (CN) starts to be heard, the locus of the SNP + verb constraint moved posteriorly into the left posterior MTG (LpMTG) and LAG which lasted until the CN is recognized. Here, we discuss our results in relation to incremental processing issues from the SNP to the CN (see Fig. 6).

### Early Activation of the SNP Constraints

Our results revealed that different aspects of SNP constraints are activated between the point at which the SNP is recognized (i.e., the UP of SN) and its offset ~100 ms later and that these computations recruit different brain areas. First, the SNP constraint on upcoming verbs (Fig. 5*a*) appeared only in mid-anterior portions of right middle/inferior temporal gyri (RMTG/ITG), whereas the SNP constraint on upcoming CNs (Fig. 5*b*) involved more extensive regions including right ATL (RATL), RIFG, and LH temporal cortex. The important similarities and differences in the neurobiological basis of these constraints are 1) the core regions involved in constructing both types of constraints, which included RH anterior MTG/ITG regions, and 2) only the nonadjacent SNP constraint on CNs elicited activation in the RIFG which lasted all the way until the verb was recognized in Epoch 2.

These regions are plausibly involved in generating and maintaining the event representations, which are naturally generated at the beginning of sentences and form a basis for semantic constraints on upcoming speech (Marslen-Wilson et al. 1993; Nieuwland and Van Berkum 2006). Various studies (Marslen-Wilson and Tyler 1980; Kamide et al. 2003) have shown that listeners use multiple sources of information at the earliest possible opportunity to establish the fullest possible interpretation of what they are hearing and demonstrate that such processes are not restricted to the syntactic structure of language. One of the prediction principles (Altmann and Mirković 2009) that underpin human language comprehension states that the mapping between the unfolding sentence and the event representation enables listeners to predict both how the language will unfold and how the real-world event will unfold, rendering prediction impossible to stand alone without incrementally developing event representations.

In line with these claims, our results revealed consistent activations of RH mid-anterior temporal regions for different semantic constraints, likely reflecting the broad scenarios activated by the SNP. This claim is further supported by three major findings from our main and complementary analyses, possibly indicating that they are activated from the same set of scenarios drawn by the SNP: 1) the same activation timing for different SNP constraints around the UP of an SN; 2) a common subspace existing between different SNP constraints (see Fig. S4 in Supplementary Materials Section 4); and 3) the joint semantic constraint of the SNP on verb and CN (i.e., the early event-level constraint) elicited a significant activity pattern in the RH mid-anterior temporal regions as well (see Fig. S5 in Supplementary Materials Section 5).

The activation of RH regions has been consistently reported when drawing coherent “message-level” interpretations in speech comprehension (Beeman and Chiarello 1998; Beeman et al. 2000; Jung-Beeman 2005), consistent with studies claiming the importance of RH in processing linguistic context (Kircher et al. 2001; Bookheimer 2002). These findings have been supported by previous ERP studies showing that the RH plays an important role in interpreting individual words with respect to a larger-scale context (Federmeier and Kutas 1999; Wlotko and Federmeier 2007; Federmeier et al. 2008), emphasizing the role of RH in processing context-driven semantic relationships (Federmeier et al. 2008). Hence, the early effect in the right temporal regions in the current study are likely related to the process of generating constraint driven by the SNP context, setting up the event-level scenarios of what is likely to be talked about (Elman 2011).

However, two additional areas in the LH temporal lobe and RIFG were engaged in constraining the nonadjacent CN based on the SNP context (Fig. 5*b*). The two critical differences between the SNP constraints are 1) the grammatical category of constrained words and 2) adjacency with respect to the SNP context. Previous studies have shown the engagement of the LH temporal regions when processing nouns compared with when processing verbs (Siri et al. 2007; Vigliocco et al. 2011).

Unlike the bilateral temporal regions, the RIFG cluster remained significant after the verb onset until the verb was recognized. Consistent with this finding, recent studies have reported RIFG as a part of the extensive network involved in constraining an upcoming word (Willems et al. 2015) and resolving semantic competition (Kocagoncu et al. 2017). More generally, this region has been involved in semantic maintenance and cognitive control (Shivde and Thompson-Schill 2004; Gajardo-Vidal et al. 2018), activating when processing an indeterminate sentence which can be interpreted in many different ways (de Almeida et al. 2016) or when encountering a word with multiple meanings in a spoken sentence (Rodd et al. 2005; Mason and Just 2007). Therefore, the SNP constraint effect in RIFG during the verb likely reflects the maintenance of the SNP semantic constraint while resolving competition as the verb is being heard.

### Evolving Constraint

The essence of incremental speech comprehension is that each word is interpreted in a context-relevant manner and the constraint derived from the prior context is updated to be more specific and informative on the upcoming words in the sentence as more words are heard (Kuperberg and Jaeger 2016). To investigate this incremental development (i.e., how the prior SNP constraint on CNs evolves as a verb is recognized), we constructed a model that captures the semantic constraint on CNs based on the full SNP + verb context. Our results showed that the effect of the SNP + verb constraint appears at 60 ms after the verb onset in the right mid-anterior MTG/ITG regions which extended to LATL and LIFG peaking around 400 ms after the verb onset (i.e., close to the mean verb offset). As the target word (i.e., CN) is being heard, the cluster moved into more posterior areas involving LMTG and LAG, which lasted until the CN is recognized (Fig. 5*c*). These transitions across time may highlight differential roles engaged by these regions when constraining the CN. For example, as discussed above, the early RH temporal effect most likely reflects the broad constraint on CN, primarily set up by the SNP (i.e., in natural language comprehension, it is highly unlikely that an incoming verb is completely incongruent with the activated scenarios). Then, the ventral fronto-temporal network in LH including LIFG (BA47/45) and LATL additionally engages in constraining the CN as the verb is recognized.

The broad scenarios activated by the SNP become more fine-tuned as the semantics of the verb is combined with the SNP context. According to the timing of LIFG-LATL activations, these regions may play an important role in resolving uncertainty by updating the sentential meaning so that it becomes more specific. Further support for this argument comes from a complementary analysis (see Fig. S3-2 and Fig. S3-3 in Supplementary Materials Section 3) showing a statistically significant reduction in entropy between the SNP constraint and the SNP + verb constraint, which reflects an important aspect of incremental speech comprehension (Hale 2006) (see Fig. 3). As LATL is directly connected to LBA47 via the uncinate fasciculus (Catani et al. 2005), our results suggest that the interaction within the anteroventral fronto-temporal network is involved in developing more informative constraint based on the combined context of SNP + verb.

After the onset of the target word (CN), we observed a significant cluster moving into more posterior regions including LpMTG and LAG until around the UP of the CN. The transition and timing of this cluster may reflect the facilitatory effect of the contextual (SNP + verb) constraint on activating semantic content of the CN as these regions are often involved in activating lexical-semantic content (Hickok and Poeppel 2007) and combining it into the preceding context at both phrasal and sentential levels (Humphries et al. 2007; Schell et al. 2017; Lyu et al. 2019). Therefore, such anterior (BA47/45 and LATL) to posterior (LpMTG/LAG) transition likely reflects the top-down (i.e., the SNP + verb constraint), bottom-up (i.e., speech input of the CN) interaction, in order to generate a coherent semantic interpretation of the CN with respect to the preceding SNP + verb context.

### Constraint Evaluation

Developing an event representation requires each word in a sentence to be interpreted in the context of the prior context. This process, in turn, requires semantically evaluating each word with respect to the prior constraint, indexed by the degree of mismatch between the context and an upcoming word. To address this issue, we tested the effect of contextual (SNP + verb) constraint on the interpretation of the target word (CN) by quantifying the degree of mismatch between the sentential context and the target word in terms of the spatiotemporal patterns of neural activity after the CN onset. We found that activity patterns in LpMTG were sensitive to the mismatch between the constrained and the actual topic representation from 370 to 520 ms (Fig. 5*d*). Interestingly, this timing occurred immediately after the constraint effect disappeared.

In the literature, LpMTG is commonly reported in studies of semantics (Price 2010) and is typically known as the source of the N400 effect (Lau et al. 2008; Kutas and Federmeier 2011). A recent study reported predictability (e.g., “runny nose” vs. “dainty nose”) estimated from corpus data modulated the N400 component in LpMTG (Lau and Namyst 2019), reducing the necessity of activating the stored lexical representation of the target word (CN in our study) when it is strongly constrained by the context (i.e., high predictability).

This argument is further supported by our previous study (Lyu et al. 2019) where the semantic representation of a CN was strongly modulated by the preceding verb; for example, the verb in context (e.g., the man “ate”) pruned the less relevant CN topics, allowing listeners to interpret the CN (e.g., “apple”) more specifically with the CN topics that were supported by the preceding verb (e.g., topics related to “food” but not those related to “shape” or “color”). While the exact computational details of the mismatch effect remain elusive, our findings suggest that listeners not only develop semantic constraints on upcoming words but they also use these constraints to efficiently derive the context-relevant interpretation of upcoming words such as the CN. Combined with other constraint effects discussed above, these results clearly illustrate the incremental stages of predictive processing that enables listeners to construct the message-level interpretation from the three crucial components in a sentence (SNP, verb, and CN).

### Implications for Future Studies

Previous studies have explained neuroimaging data using computational models to quantify entropy at lexical (Frank et al. 2015; Willems et al. 2015) and phonological levels (Donhauser and Baillet 2020). In these studies, neural network models with a recurrent architecture were commonly employed to generate a context-dependent linguistic prediction as a probability distribution from which entropy can be computed. On top of these studies, the current study examined the semantic aspect of incremental language prediction using entropy of topic distributions, designed to express the co-occurrence relation among words in different grammatical categories through estimating the expected posterior of the multinomial parameters (see Supplementary Material Section 1). In this section, we motivate the choice of our computational model and approach while discussing its limitations and directions for future studies.

Recent advances in the field of computer science have established a number of different computational algorithms to construct distributional semantic models, optimally reflecting the content of each lexical item in a set of latent dimensions. Perhaps, the currently most popular algorithm is the neural network training with a recurrent architecture, including recurrent neural network (RNN) and long short-term memory (LSTM). However, we chose to use topic modeling based on LDA to exploit its two critical aspects.

1. It produces a semantic vector of a word as a probability distribution over latent semantic dimensions (topics). This allows us to construct our incremental models under the Bayesian computational framework (Kuperberg and Jaeger 2016), a useful approach for understanding predictive processing in language.
2. It explicitly depicts the semantic relations between words in different positions in a sentence. Our implementation of topic modeling, which treats SNs and verbs as “documents” and CNs as “words,” is specifically designed to explain semantic prediction and updates based on key words in the context.

Its explanatory value as a predictive model is one of its biggest assets, making it particularly attractive in the field of psycho- and neuro-linguistics. Nonetheless, one critical limitation of this approach is that it is not an incremental model by itself, unlike RNN or LSTM. To address this issue, we introduced the method of blending a set of topic vectors based on Cloze probabilities calculated from sentence completion studies.

Despite the popularity of Cloze probability as a direct behavioral measure of human prediction, its application entails high subjective bias, often affected by confounding factors such as familiarity (Smith and Levy 2011). Although Cloze probability was significantly related to corpus probability, it also significantly deviated from the corpus probability with greater entropy in responses, making Cloze a suboptimal estimate of linguistic prediction which has been successful in explaining neural responses (DeLong et al. 2005; Kutas and Federmeier 2011). Moreover, another confounding factor of Cloze is that the prediction may well be driven by a pragmatic inferential process, not purely by semantic associations. Hence, it remains controversial whether the basis of the incremental prediction is semantic or pragmatic in nature. Despite the objective and accurate probability estimates that large-scale corpora offer, there is a practical limitation of applying the corpus probability as the number of words increases in the model (i.e., increasing *N* in an *N*-gram probability). Even with large-scale corpora, the estimation of co-occurrence probability becomes very difficult with *N* > 3. With our stimuli containing 6–7 words before the CN (e.g., “The experienced walker chose the path”), computing a conditional probability becomes impossible.

Taken together, future studies need to develop a self-explanatory incremental model, allowing us to characterize evolving representations. Recent developments of more sophisticated models such as generative pretraining (Radford et al. 2018) have shown impressive performance on making output predictions, but their multilayered internal representations are highly complex and lack an explanatory value to provide insights into predictive processing in the human brain. Quantifying different aspects of representation that incrementally evolve over time in these models will initiate more model-driven decoding research on brain data, shedding light on the neurobiological basis of incremental speech comprehension. At last, although we constrained our search space within a language mask to characterize linguistic aspects of predictive processing and specific computations involved in constraining upcoming words, other brain networks involved in different cognitive functions, such as attention and/or memory, may also be involved in such linguistic processes of understanding speech. With the ultimate goal of expanding our research to discourse and narrative comprehension, such whole-brain analysis will contribute to understanding the interactive nature of cognitive processes during language comprehension.

Finally, there have been growing efforts to elucidate the interactive nature of cognition, bringing multiple domains of cognition such as language and memory into a unifying framework (Duff and Brown-Schmidt 2017). For example, developing an event representation involves the episodic realization (e.g., “orange” in “She peeled an orange, and ate it quickly”) of a semantic type (e.g., “orange” in general). The role of such episodic-semantic interface during natural language comprehension is extensively discussed in a recent account (Altmann 2017), claiming the hippocampal structures as one of the neurobiological bases for encoding distinct episodes (McClelland et al. 1995). While we have shown that incremental predictive processes can be characterized even with such generic linguistic stimuli, we advocate the need for more specific stimuli in a narrative context in order to distinguish an episodic token from a semantic type. In this way, the stimuli would have sufficient variability to provide the distinguishable representational geometry between them, allowing researchers to investigate the interactive event dynamics beyond combinatorial semantics from semantic memory alone.

As a final remark, this study focused on presenting a possible approach to investigate one of the core processes (i.e., prediction) of human event cognition during natural speech comprehension. Future studies will need to expand this research to investigate other central cognitive processes involved in understanding the event dynamics and illuminate its neurobiological underpinnings, likely recruiting multiple interactive networks in the brain outside the language network.

## Conclusion

In this study, we demonstrated the neurobiological basis of incremental predictive language processing by characterizing the spatiotemporal dynamics of source-localized EMEG data with ssRSA using rich co-occurrence computational semantic models based on topic modeling combined with human behavioral data.

To summarize our results, an extensive bilateral fronto-temporo-parietal network is actively engaged in generating and developing incremental semantic constraints on upcoming words (see Fig. 6). Our results highlight the temporal progression of semantic constraint development: 1) an RH fronto-temporal network initially generates possible scenarios as the SNP is heard which, in turn, 2) recruits a LH fronto-temporal network as the scenarios get enriched as subsequent words are heard (a verb in this case), and (3) terminating in a LH posterior temporo-parietal network as the target word (CN) is recognized. To our knowledge, none of the neurobiological models of speech comprehension have explained this range of sequential temporal relationships among multiple regions in the language network during incremental speech comprehension, largely due to the lack of evidence for characterizing the spatiotemporal dynamics of neural activity. Further research is needed to understand the detailed neural mechanisms underpinning these important effects.

## Notes

We thank Dr Barry Devereux for his help in the early stages of this research.

## Funding

European Research Council Advanced Investigator Grant to L.K.T. under the European Community’s Horizon 2020 Research and Innovation Programme (2014-2020 ERC Grant Agreement 669820).

## Supplementary Material

CerCor20200002551_SI_section_1_bhaa222

CerCor20200002551_SI_section_2_bhaa222

CerCor20200002551_SI_section_3_bhaa222

CerCor20200002551_SI_section_4_bhaa222

CerCor20200002551_SI_section_5_bhaa222

## References

[ref1] Altmann GTM 2017 Abstraction and generalization in statistical learning: implications for the relationship between semantic types and episodic tokens. Philos Trans R Soc B Biol Sci. 372: 20160060. 10.1098/rstb.2016.0060 PMC5124085 27872378

[ref2] Altmann GTM, Mirković J 2009 Incrementality and prediction in human sentence processing. Cognit Sci. 33:583–609. 20396405 10.1111/j.1551-6709.2009.01022.x PMC2854821

[ref3] Baayen RH, Piepenbrock R, Van Rijn H 1993 The CELEX lexical database (CD-ROM). Linguistic data consortium. Philadelphia (PA): University of Pennsylvania.

[ref4] Baroni M, Lenci A 2010 Distributional memory: a general framework for corpus-based semantics. Comput Linguist. 36:673–721.

[ref5] Beeman MJ, Bowden EM, Gernsbacher MA 2000 Right and left hemisphere cooperation for drawing predictive and coherence inferences during normal story comprehension. Brain Lang. 71:310–336. 10716864 10.1006/brln.1999.2268 PMC4364278

[ref6] Beeman MJ, Chiarello C 1998 Complementary right-and left-hemisphere language comprehension. Curr Dir Psychol Sci. 7:2–8.

[ref7] Bicknell K, Elman JL, Hare M, McRae K, Kutas M 2010 Effects of event knowledge in processing verbal arguments. J Mem Lang. 63:489–505. 21076629 10.1016/j.jml.2010.08.004 PMC2976562

[ref8] Binder JR, Desai RH, Graves WW, Conant LL 2009 Where is the semantic system? A critical review and meta-analysis of 120 functional neuroimaging studies. Cereb Cortex. 19:2767–2796. 19329570 10.1093/cercor/bhp055 PMC2774390

[ref9] Bookheimer S 2002 Functional MRI of language: new approaches to understanding the cortical organization of semantic processing. Annu Rev Neurosci. 25:151–188. 12052907 10.1146/annurev.neuro.25.112701.142946

[ref10] Bornkessel-Schlesewsky I, Schlesewsky M 2013 Reconciling time, space and function: a new dorsal–ventral stream model of sentence comprehension. Brain Lang. 125:60–76. 23454075 10.1016/j.bandl.2013.01.010

[ref11] Campbell KL, Tyler LK 2018 Language-related domain-specific and domain-general systems in the human brain. Curr Opin Behav Sci. 21:132–137. 30057936 10.1016/j.cobeha.2018.04.008 PMC6058087

[ref12] Catani M, Jones DK, Ffytche DH 2005 Perisylvian language networks of the human brain. Ann Neurol Off J Am Neurol Assoc Child Neurol Soc. 57:8–16. 10.1002/ana.20319 15597383

[ref13] de Almeida RG, Riven L, Manouilidou C, Lungu O, Dwivedi VD, Jarema G, Gillon B 2016 The neuronal correlates of indeterminate sentence comprehension: an fMRI study. Front Hum Neurosci. 10:614. 28066204 10.3389/fnhum.2016.00614 PMC5168646

[ref14] DeLong KA, Troyer M, Kutas M 2014 Pre-processing in sentence comprehension: sensitivity to likely upcoming meaning and structure. Lang Linguist Compass. 8:631–645. 27525035 10.1111/lnc3.12093 PMC4982702

[ref15] DeLong KA, Urbach TP, Kutas M 2005 Probabilistic word pre-activation during language comprehension inferred from electrical brain activity. Nat Neurosci. 8:1117. 16007080 10.1038/nn1504

[ref16] Donhauser PW, Baillet S 2020 Two distinct neural timescales for predictive speech processing. Neuron. 105:385–393. 31806493 10.1016/j.neuron.2019.10.019 PMC6981026

[ref17] Duff MC, Brown-Schmidt S 2017 Hippocampal contributions to language use and processing In: Hannula D, Duff M, editors. The hippocampus from cells to systems. Springer International Publishing, pp. 503–536.

[ref18] Elman JL 2011 Lexical knowledge without a lexicon? Ment Lex. 6:1–33. 22069438 10.1075/ml.6.1.01elm PMC3209550

[ref19] Federmeier KD, Kutas M 1999 Right words and left words: electrophysiological evidence for hemispheric differences in meaning processing. Cogn Brain Res. 8:373–392. 10.1016/s0926-6410(99)00036-1 10556614

[ref20] Federmeier KD, Wlotko EW, Meyer AM 2008 What’s ‘Right’in language comprehension: event-related potentials reveal right hemisphere language capabilities. Lang Linguist Compass. 2:1–17. 19777128 10.1111/j.1749-818X.2007.00042.x PMC2748422

[ref21] Frank SL, Otten LJ, Galli G, Vigliocco G 2015 The ERP response to the amount of information conveyed by words in sentences. Brain Lang. 140:1–11. 25461915 10.1016/j.bandl.2014.10.006

[ref22] Friederici AD 2011 The brain basis of language processing: from structure to function. Physiol Rev. 91:1357–1392. 22013214 10.1152/physrev.00006.2011

[ref23] Gajardo-Vidal A, Lorca-Puls DL, Hope TMH, Parker Jones O, Seghier ML, Prejawa S, Crinion JT, Leff AP, Green DW, Price CJ 2018 How right hemisphere damage after stroke can impair speech comprehension. Brain. 141:3389–3404. 30418586 10.1093/brain/awy270 PMC6262220

[ref24] Griffiths TL, Steyvers M 2004 Finding scientific topics. Proc Natl Acad Sci. 101:5228–5235. 14872004 10.1073/pnas.0307752101 PMC387300

[ref25] Hagoort P 2013 MUC (memory, unification, control) and beyond. Front Psychol. 4:416. 23874313 10.3389/fpsyg.2013.00416 PMC3709422

[ref26] Hagoort P, Baggio G, Willems RM 2009 Semantic unification In: Gazzaniga M, editor. The cognitive neurosciences. 4th ed. Cambridge, Mass, USA: MIT Press, pp. 819–836.

[ref27] Hale J 2001 A probabilistic Earley parser as a psycholinguistic model In: Proceedings of the second meeting of the North American Chapter of the Association for Computational Linguistics on Language technologies. Association for Computational Linguistics p. 1–8.

[ref28] Hale J 2006 Uncertainty about the rest of the sentence. Cognit Sci. 30:643–672. 21702829 10.1207/s15516709cog0000_64

[ref29] Hickok G, Poeppel D 2007 The cortical organization of speech processing. Nat Rev Neurosci. 8:393. 17431404 10.1038/nrn2113

[ref30] Humphries C, Binder JR, Medler DA, Liebenthal E 2007 Time course of semantic processes during sentence comprehension: an fMRI study. Neuroimage. 36:924–932. 17500009 10.1016/j.neuroimage.2007.03.059 PMC1941617

[ref31] Jung-Beeman M 2005 Bilateral brain processes for comprehending natural language. Trends Cogn Sci. 9:512–518. 16214387 10.1016/j.tics.2005.09.009

[ref32] Kamide Y, Altmann GTM, Haywood SL 2003 The time-course of prediction in incremental sentence processing: evidence from anticipatory eye movements. J Mem Lang. 49:133–156.

[ref33] Kircher TTJ, Brammer M, Andreu NT, Williams SCR, McGuire PK 2001 Engagement of right temporal cortex during processing of linguistic context. Neuropsychologia. 39:798–809. 11369403 10.1016/s0028-3932(01)00014-8

[ref34] Klimovich-Gray A, Tyler LK, Randall B, Kocagoncu E, Devereux B, Marslen-Wilson WD 2019 Balancing prediction and sensory input in speech comprehension: the spatiotemporal dynamics of word recognition in context. J Neurosci. 39:519–527. 30459221 10.1523/JNEUROSCI.3573-17.2018 PMC6335748

[ref35] Kocagoncu E, Clarke A, Devereux BJ, Tyler LK 2017 Decoding the cortical dynamics of sound-meaning mapping. J Neurosci. 37:1312–1319. 28028201 10.1523/JNEUROSCI.2858-16.2016 PMC6596862

[ref36] Korhonen A, Krymolowski Y, Briscoe T 2006 A Large Subcategorization Lexicon for Natural Language Processing Applications In: LREC. p. 1015–1020.

[ref37] Kuperberg GR 2016 Separate streams or probabilistic inference? What the N400 can tell us about the comprehension of events. Lang Cogn Neurosci. 31:602–616. 27570786 10.1080/23273798.2015.1130233 PMC4996121

[ref38] Kuperberg GR, Jaeger TF 2016 What do we mean by prediction in language comprehension? Lang Cogn Neurosci. 31:32–59. 27135040 10.1080/23273798.2015.1102299 PMC4850025

[ref39] Kutas M, Federmeier KD 2011 Thirty years and counting: finding meaning in the N400 component of the event-related brain potential (ERP). Annu Rev Psychol. 62:621–647. 20809790 10.1146/annurev.psych.093008.131123 PMC4052444

[ref40] Lau EF, Namyst A 2019 fMRI evidence that left posterior temporal cortex contributes to N400 effects of predictability independent of congruity. Brain Lang. 199:104697. 31585316 10.1016/j.bandl.2019.104697

[ref41] Lau EF, Phillips C, Poeppel D 2008 A cortical network for semantics:(de) constructing the N400. Nat Rev Neurosci. 9:920. 19020511 10.1038/nrn2532

[ref42] Levy R 2008 Expectation-based syntactic comprehension. Cognition. 106:1126–1177. 17662975 10.1016/j.cognition.2007.05.006

[ref43] López JD, Litvak V, Espinosa JJ, Friston K, Barnes GR 2014 Algorithmic procedures for Bayesian MEG/EEG source reconstruction in SPM. Neuroimage. 84:476–487. 24041874 10.1016/j.neuroimage.2013.09.002 PMC3913905

[ref44] Luke SG, Christianson K 2016 Limits on lexical prediction during reading. Cogn Psychol. 88:22–60. 27376659 10.1016/j.cogpsych.2016.06.002

[ref45] Lyu B, Choi HS, Marslen-Wilson WD, Clarke A, Randall B, Tyler LK 2019 Neural dynamics of semantic composition. Proc Natl Acad Sci. 116:21318–21327. 31570590 10.1073/pnas.1903402116 PMC6800340

[ref46] Maris E, Oostenveld R 2007 Nonparametric statistical testing of EEG-and MEG-data. J Neurosci Methods. 164:177–190. 17517438 10.1016/j.jneumeth.2007.03.024

[ref47] Marslen-Wilson WD 1975 Sentence perception as an interactive parallel process. Science (80- ). 189:226–228. 10.1126/science.189.4198.226 17733889

[ref48] Marslen-Wilson WD 1987 Functional parallelism in spoken word-recognition. Cognition. 25:71–102. 3581730 10.1016/0010-0277(87)90005-9

[ref49] Marslen-Wilson WD, Tyler LK 1980 The temporal structure of spoken language understanding. Cognition. 8:1–71. 7363578 10.1016/0010-0277(80)90015-3

[ref50] Marslen-Wilson WD, Tyler LK 2007 Morphology, language and the brain: the decompositional substrate for language comprehension. Philos Trans R Soc B Biol Sci. 362:823–836. 10.1098/rstb.2007.2091 PMC2430000 17395577

[ref51] Marslen-Wilson WD, Tyler LK, Koster C 1993 Integrative processes in utterance resolution. J Mem Lang. 32:647–666.

[ref52] Mason RA, Just MA 2007 Lexical ambiguity in sentence comprehension. Brain Res. 1146:115–127. 17433891 10.1016/j.brainres.2007.02.076 PMC2713009

[ref53] Matchin W, Hickok G 2020 The cortical organization of syntax. *Cereb Cortex*. 30:1481–1498. 10.1093/cercor/bhz180 PMC7132936 31670779

[ref54] Mattout J, Henson RN, Friston KJ 2007 Canonical source reconstruction for MEG. Comput Intell Neurosci. 2017:67613. 10.1155/2007/67613 PMC2266807 18350131

[ref55] McClelland JL, McNaughton BL, O’Reilly RC 1995 Why there are complementary learning systems in the hippocampus and neocortex: insights from the successes and failures of connectionist models of learning and memory. Psychol Rev. 102:419. 7624455 10.1037/0033-295X.102.3.419

[ref56] Nieuwland MS, Van Berkum JJA 2006 When peanuts fall in love: N400 evidence for the power of discourse. J Cogn Neurosci. 18:1098–1111. 16839284 10.1162/jocn.2006.18.7.1098

[ref57] Price CJ 2010 The anatomy of language: a review of 100 fMRI studies published in 2009. Ann N Y Acad Sci. 1191:62–88. 20392276 10.1111/j.1749-6632.2010.05444.x

[ref58] Price CJ 2012 A review and synthesis of the first 20 years of PET and fMRI studies of heard speech, spoken language and reading. Neuroimage. 62:816–847. 22584224 10.1016/j.neuroimage.2012.04.062 PMC3398395

[ref59] Radford A, Narasimhan K, Salimans T, Sutskever I. 2018 Improving language understanding by generative pre-training. https//s3-us-west-2 Amaz com/openai-assets/researchcovers/languageunsupervised/language Underst Pap pdf

[ref60] Rodd JM, Davis MH, Johnsrude IS 2005 The neural mechanisms of speech comprehension: fMRI studies of semantic ambiguity. Cereb Cortex. 15:1261–1269. 15635062 10.1093/cercor/bhi009

[ref61] Schell M, Zaccarella E, Friederici AD 2017 Differential cortical contribution of syntax and semantics: an fMRI study on two-word phrasal processing. Cortex. 96:105–120. 29024818 10.1016/j.cortex.2017.09.002

[ref62] Seger CA, Desmond JE, Glover GH, Gabrieli JDE 2000 Functional magnetic resonance imaging evidence for right-hemisphere involvement in processing unusual semantic relationships. Neuropsychology. 14:361. 10928739 10.1037//0894-4105.14.3.361

[ref63] Shivde G, Thompson-Schill SL 2004 Dissociating semantic and phonological maintenance using fMRI. Cogn Affect Behav Neurosci. 4:10–19. 15259886 10.3758/cabn.4.1.10

[ref64] Siri S, Tettamanti M, Cappa SF, Della RP, Saccuman C, Scifo P, Vigliocco G 2007 The neural substrate of naming events: effects of processing demands but not of grammatical class. Cereb Cortex. 18:171–177. 17507455 10.1093/cercor/bhm043

[ref65] Smith N, Levy R 2011 Cloze but no cigar: the complex relationship between cloze, corpus, and subjective probabilities in language processing In: Proceedings of the Annual Meeting of the Cognitive Science Society.

[ref66] St George M, Kutas M, Martinez A, Sereno MI 1999 Semantic integration in reading: engagement of the right hemisphere during discourse processing. Brain. 122:1317–1325. 10388797 10.1093/brain/122.7.1317

[ref67] Su L, Fonteneau E, Marslen-Wilson W, Kriegeskorte N 2012 Spatiotemporal searchlight representational similarity analysis in EMEG source space In: 2012 Second International Workshop on Pattern Recognition in NeuroImaging. IEEE p. 97–100.

[ref68] Taulu S, Simola J 2006 Spatiotemporal signal space separation method for rejecting nearby interference in MEG measurements. Phys Med Biol. 51:1759. 16552102 10.1088/0031-9155/51/7/008

[ref69] Tyler LK, Marslen-Wilson WD 1977 The on-line effects of semantic context on syntactic processing. J Verbal Learning Verbal Behav. 16:683–692.

[ref70] Vigliocco G, Vinson DP, Druks J, Barber H, Cappa SF 2011 Nouns and verbs in the brain: a review of behavioural, electrophysiological, neuropsychological and imaging studies. Neurosci Biobehav Rev. 35:407–426. 20451552 10.1016/j.neubiorev.2010.04.007

[ref71] Willems RM, Frank SL, Nijhof AD, Hagoort P, Van den Bosch A 2015 Prediction during natural language comprehension. Cereb Cortex. 26:2506–2516. 25903464 10.1093/cercor/bhv075

[ref72] Wlotko EW, Federmeier KD 2007 Finding the right word: hemispheric asymmetries in the use of sentence context information. Neuropsychologia. 45:3001–3014. 17659309 10.1016/j.neuropsychologia.2007.05.013 PMC2066191

[ref73] Wright P, Stamatakis EA, Tyler LK 2012 Differentiating hemispheric contributions to syntax and semantics in patients with left-hemisphere lesions. J Neurosci. 32:8149–8157. 22699896 10.1523/JNEUROSCI.0485-12.2012 PMC3575031

