# Supplementary material for: Decoding the Real-Time Neurobiological Properties of Incremental Semantic Interpretation
Source: Cereb Cortex. 2020 Aug 31;31(1):233–47. doi: 10.1093/cercor/bhaa222 (PMC7727355; doi:10.1093/cercor/bhaa222)
Supplement: CerCor20200002551_SI_section_1_bhaa222 [file cercor20200002551_si_section_1_bhaa222.docx]

**Supplementary Information**

**SI section 1: Topic modelling with Latent Dirichlet Allocation**

Modelling the semantics of a word involves taking into account the linguistic context in which it occurs. Unlike feature-based conceptual semantic models (Tyler and Moss 2001), co-occurrence based semantic models naturally reflect the statistical relations among different words based on the fundamental assumption that semantically similar words appear in similar contexts (Harris 1954). Such co-occurrence based semantic models enable semantic contents to be induced from the statistics of large-scale text corpora. Hence, they provide rich distributional content for every word, encoded in the multi-dimensional semantic space whose geometric location and relative distance from the other words define its semantic identity. Such well-defined representational properties allow us to develop reliable models of semantic computations, using quantifiable measures that effectively summarise the representation (or semantic content).

Latent Dirichlet Allocation (LDA) is one of the distributional semantic modelling (DSM) approaches to express the co-occurrence relations in a latent semantic space, assigning every word to one or more latent dimensions in a way that maximises the posterior probability of the model. Just like any other DSM approach, it is built upon the distributional hypothesis which claims that any words that occur in similar (linguistic) context are semantically similar (Harris 1954). Its distinct quality is in its formulation in a probabilistic (Bayesian) framework that takes the advantage of using a Dirichlet prior, the conjugate distribution of the multinomial likelihood. In this way, it is possible to marginalize the parameter(s) and express the Dirichlet posterior in terms of the known variable (i.e. observed samples) and the hyper-parameter(s).

The model training involves iterative updating of two probability distributions, known as target-topic (e.g. $P\left( target\_word | topic \right)$) and topic-context (e.g. $P(topic|context\_word)$) distributions:

$$P\left( target\_word | context\_word \right)=\sum_{topic} P\left( target\_word | topic \right)P(topic|context\_word)$$

Each of these distributions was parameterized by separate multinomial variables (e.g. $\emptyset$ and $\theta$) that specify a distribution either over target words for a given topic (target-topic), or over topics for a given context (topic-context). Then, using collapsed Gibbs sampler, we computed the maximum a posterior (MAP) estimate of each of these parameters as the following:

$$E_{pos}\left[ \emptyset_{j,w_{i}} \right]=P\left( w_{i} | z_{i}=j,z_{-i},w_{-i} \right)=\frac{f_{-i,j}^{(w_{i})}+\beta}{f_{-i,j}+|W|\beta}$$

$$E_{pos}\left[ \theta_{c_{i},j} \right]=P\left( z_{i}=j | z_{-i},c_{i} \right)=\frac{f_{-i,j}^{(c_{i})}+\alpha_{j}}{\sum_{j} f_{-i,j}^{(c_{i})}+\alpha_{j}}$$

where $i$ and $j$ are indices to observed samples and latent dimensions (topics) respectively, $w_{i}$, $c_{i}$ and $z_{i}$ are the $target\_word$, $context\_word$ and $topic$ at the $i$^th^ (current) observation, $-i$ represents all observations other than the $i$^th^ observation, $\beta$ and $\alpha_{j}$ are the symmetric and asymmetric hyper-parameters (Wallach et al. 2009) associated with $\emptyset$ and $\theta$ respectively,$|W|$ is the total number of words in the vocabulary and $f$ represents the frequency count such that $f_{-i,j}^{(w_{i})}$ is the frequency of a word at the current observation $i$ associated with the topic $j$ after taking out a topic assignment at $i$. The hyper-parameters were optimised in a way that maximises the model evidence using the fixed-point iteration scheme (Minka 2000).

For the actual model training, the two hyper-parameters were initially randomized but were jointly updated for every observation in the training dataset by re-sampling the topic after taking out the randomly assigned topic and computing the leave-one-out probability distributions. This training approach is known as collapsed Gibbs sampler (a variant of Gibbs sampling that involves marginalization of the multinomial parameters), one of the well-known Markov chain Monte Carlo (MCMC) methods which obtain a sample by observing the chain (whose desired distribution is same as its equilibrium distribution) after a number of training steps. Here, each chain refers to a stochastic model at a given training step whose probability distributions are computed based solely on the previous step such that the future state of the system is conditionally independent to its past states given its present state. Not surprisingly, the random sample (or a state of the model at each step) from this MCMC method is inherently auto-correlated. Therefore, we took the distributions from three sampling states which were 50 training steps apart from each other (to maximize the degree of independence between sampling states) after the burn-in period of 200 steps (for model stabilization). The total number of topics was set to 100 ($\left| Z \right|=100$). All these parameters were set to be consistent with (Ó Séaghdha and Korhonen 2014). The distributions from these samples were averaged and the averaged topic-context distribution was used as a model of semantic representation. See Figure S1 for an illustration of the explanatory values of our topic model.


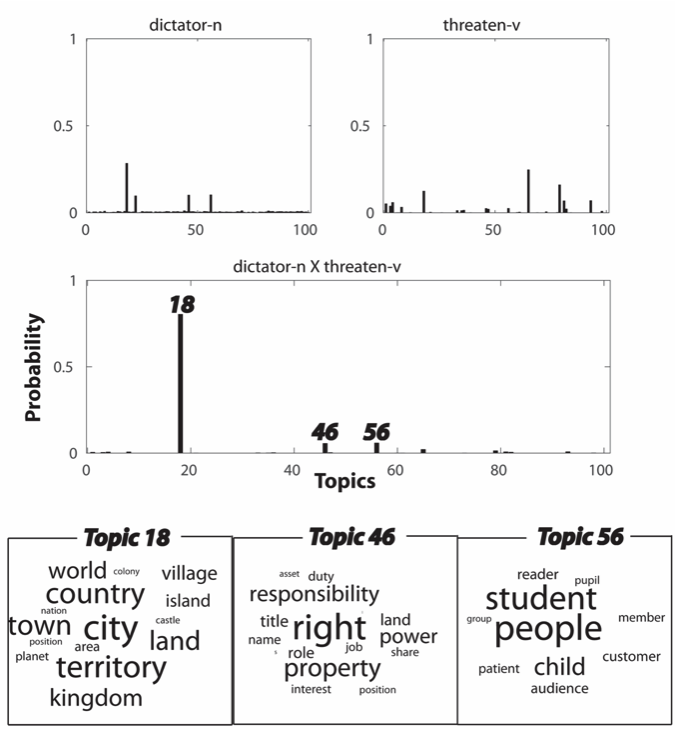


Figure S1: *A visual illustration of our SNV-CN topic model. In the top row, the predictive activations of “dictator” (SN) and “threaten” (verb) are depicted by our topic-context distributions. In the middle row, we model the consistency in topic preference between “dictator” and “threaten” using an element-wise multiplication (.*) as a combinatorial operator (re-normalised for visualisation). Lastly, in the bottom row, a list of complement nouns (CNs) preferred by each of the top three consistent topics is visualised (The size of each CN in the list reflects the target-topic probability from our topic model).*

**References**

Harris ZS. 1954. Distributional structure. Word. 10:146–162.

Minka T. 2000. Estimating a Dirichlet distribution.

Ó Séaghdha D, Korhonen A. 2014. Probabilistic distributional semantics with latent variable models. Comput Linguist. 40:587–631.

Tyler LK, Moss HE. 2001. Towards a distributed account of conceptual knowledge. Trends Cogn Sci. 5:244–252.

Wallach HM, Mimno DM, McCallum A. 2009. Rethinking LDA: Why priors matter. In: Advances in neural information processing systems. p. 1973–1981.
