# Supplementary material for: Decoding the Real-Time Neurobiological Properties of Incremental Semantic Interpretation
Source: Cereb Cortex. 2020 Aug 31;31(1):233–47. doi: 10.1093/cercor/bhaa222 (PMC7727355; doi:10.1093/cercor/bhaa222)
Supplement: CerCor20200002551_SI_section_2_bhaa222 [file cercor20200002551_si_section_2_bhaa222.docx]

**Supplementary Information**

**SI section 2: Effect size analysis**

In this section, we report the results of our effect size analysis, showing that all of our results presented in the main text are reliable. We carried out an effect size analysis where we calculated an effect size map (i.e. Cohen’s D) and a power map based on $P\left( t_{obs}-t_{crit}>0 \right)$ where $t_{obs}$ is the observed t-value for every data-point (searchlight) across space and time and $t_{crit}$ is the critical t-value determined by the false positive rate (alpha) = 0.05. For statistical summary, we computed an average effect size (D) and power across all searchlights within a cluster reported in the main text (Figure 5).

Overall, our results clearly showed a strong effect size (>0.8) and a reliable statistical power (~90%) for all of the clusters reported in this study (see Figure S2) (compare Figure S2 with Figure 5 in the main text). The mismatch analysis at Epoch 3 based on 128 items (stimuli) had the lowest, yet strong effect size and power (see panel (d) in Figure S2). This is likely because we used a stringent cluster-forming threshold (CFT: p=.01 instead of p=.05) which compensates for the modest sample size (N=13). Based on these findings, we claim that the reported effects are strong and reliable.


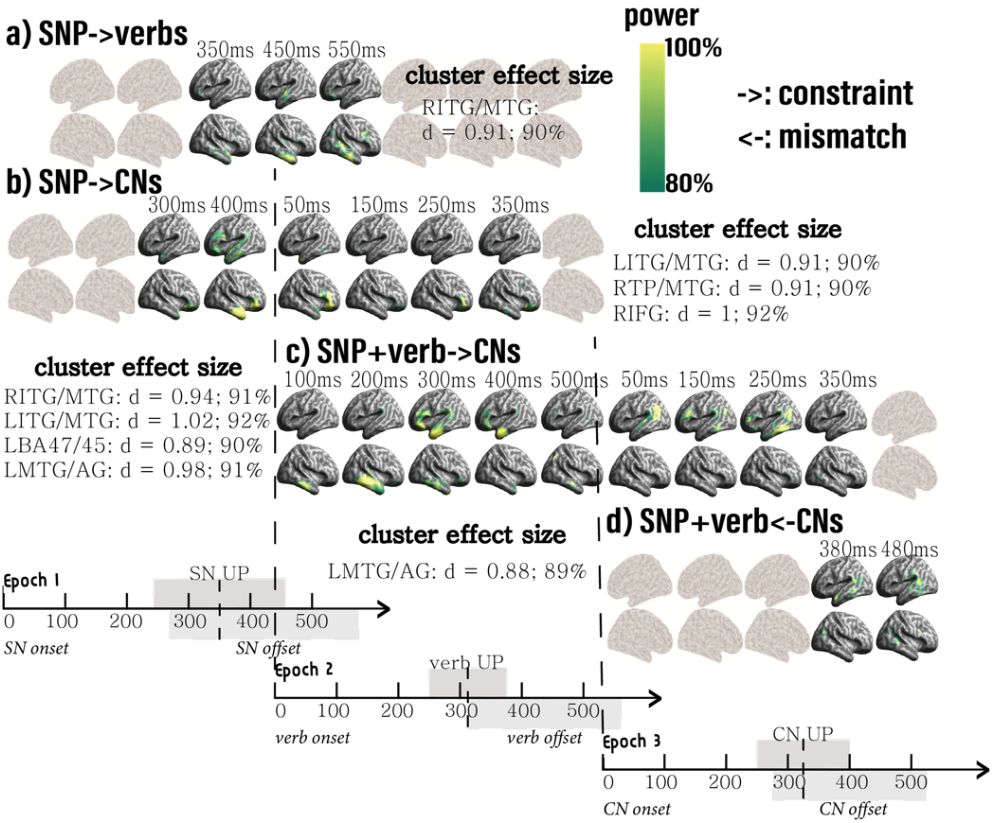


Figure S2: *Results of the power analysis with the current sample size N = 13. An additional power analysis was conducted for all of the results presented in Figure 5 in the main text at the same epochs. For visualization, we rendered any vertices above 80% power threshold but the cluster effect size in each of the four panels shows the effect size statistics (Cohen’s d) and the power of our t-statistics averaged over all searchlights within each cluster reported in the main text.*
