# Supplementary material for: Decoding the Real-Time Neurobiological Properties of Incremental Semantic Interpretation
Source: Cereb Cortex. 2020 Aug 31;31(1):233–47. doi: 10.1093/cercor/bhaa222 (PMC7727355; doi:10.1093/cercor/bhaa222)
Supplement: CerCor20200002551_SI_section_3_bhaa222 [file cercor20200002551_si_section_3_bhaa222.docx]

**Supplementary Information**

**SI section 3: Entropy reduction (evolving constraint) analysis**

If the anteroventral network (LIFG-LATL) subserves the integrative process of unifying the verb into the SNP (see (c) in Results), they essentially will lead to a reduction in uncertainty by pruning any irrelevant topics based on the verb. In order to demonstrate this uncertainty reduction in our stimuli, we statistically compared the entropy values of the semantic blends before and after the verb by running a paired-sample t-test (i.e. we statistically compared the two entropy models which were described in 3.4-2) and 3.4-3) in Methods and reported in b) and c) in Results). Consistent with our prediction, we found significant reduction in entropy after adding a verb to the context (t(45)=7.4, p<.001). See Figure S3-1 for further descriptive statistics.


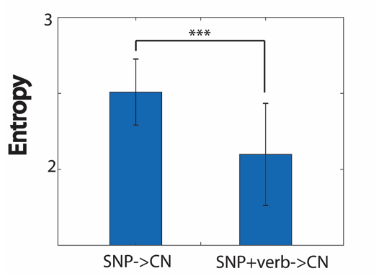


Figure S3-1: *Description of mean and standard deviation of entropy before and after integrating a verb into the SNP context. *** indicates significant difference (p<.001).*

Following this analysis, we further constructed the entropy change model which captures how specific the constraint has become by integrating a verb into the context. It was simply calculated as the amount of reduction in entropy after integrating a verb into the SNP context:

$$H\left[ P\left( CN topic | SNP \right) \right]-H[P\left( CN topic | SNP+verb \right)]$$

In order to investigate how semantic constraint is neurally evolved, this model was tested in Epoch 2 and 3 using the exactly same ssRSA analysis pipeline as described in the Methods section 4.

To our surprise, we found a significant cluster not in the LIFG-LATL regions but in the RH mid-anterior temporal regions from 180ms to 510ms in Epoch 2 (p=.016; see panel (a) in Figure S3-2). This may suggest the role of the RH mid-anterior temporal areas in both early construction and computational development of the context-based semantic constraint during natural speech comprehension. However, it does not fully explain the role of LIFG-LATL areas during incremental predictive processing.

Based on our previous findings (Lyu et al. 2019) which showed the verb-CN interaction effect in LIFG (i.e. integrated semantic representation above and beyond the semantic representation of individual words), we hypothesized that combinatorial processing of lexically-based constraints primarily recruits LIFG, projecting to the LH temporal cortex via ventral pathways (e.g. uncinate fasciculus and extreme capsule). When constraining CNs, the preceding SN and verb are the two most important words, setting up a scene for an upcoming CN. Hence, it is often very difficult to fully separate the context-based from words-based constraints. In this study, we tested an additional entropy change model calculated as: $H\left[ P\left( CN topic | SN \right) \right]-H[P\left( CN topic | SN+verb \right)]$. $P\left( CN topic | SN \right)$ was taken directly from our second SNV-CN topic model (see 3.2 in Methods) and $P\left( CN topic | SN+verb \right)$ was calculated as an element-wise multiplication between the SN-topic and verb-topic distributions:

$$P\left( CN topic | SN+verb \right)=P\left( CN topic | SN \right).*P(CN topic|verb)$$

which was normalized into a probability scale.

Testing this word-based entropy reduction model yielded a significant effect in LIFG-LATL areas, but at the later stage while hearing the CN approximately until its offset (L-BA45/47: p=.011 and LITG/MTG: p=.032; see panel (b) in Figure S3-2). This likely reflects the ongoing fine-tuning of the constraint while processing the bottom-up input. In addition, there was a hint of such computation earlier in LIFG from 230ms to 420ms in Epoch 2 which did not reach the significance threshold after the multiple comparisons correction (L-BA45: p=.09).

To further clarify if this lexically based entropy change model accounts for the LIFG-LATL clusters of the full-context SNP+verb constraint on CNs, we carried out an additional statistical analysis where we added this entropy change model as a convariate and partialled out when correlating the SNP+verb constraint model RDM with the searchlight data RDMs. Then, the output correlation map was 1) statistically tested against zero in the same way as described in the Methods section 8, and 2) statistically compared with the original correlation map without adding the entropy change model as a covariate. For this purpose, we used a one-tailed paired-sample t-test, testing against a null hypothesis that there is no difference between the two correlation maps, paired across subjects. Only for this contrast analysis, we specified the contrast window from 230ms to 460ms (the time window during which significant clusters of SNP+verb constraint on CNs emerged in LIFG-LATL areas (see panel (c) in Figure 5)). These two statistical analyses were conducted after the verb-onset in Epoch 2.

First, we found two clusters of the SNP+verb constraint on CNs in the bilateral temporal regions from 50ms to 460ms after the verb-onset when lexically-based entropy change was partialled out (p=.002 in RITG/MTG and p=.047 in LITG/MTG; see panel (a) in Figure S3-3). Second, we showed that the SNP+verb constraint model fit is significantly reduced in LIFG from 240ms to 450ms after the verb-onset (p=.031 in L-BA45; see panel (b) in Figure S3-3). Combining these results, we suggest that the process of integrating semantic constraint from multiple lexical sources recruits L-BA45/47 which interactively develops the context-level constraint to become more specific. Although this is highly consistent with previous findings and neurobiological accounts of speech comprehension (Hagoort 2013; Kocagoncu et al. 2017; Lyu et al. 2019), future studies that investigate the spatiotemporal neural dynamics during incremental sentence/discourse comprehension will further establish the role of multiple brain regions in the language network beyond lexical processing.


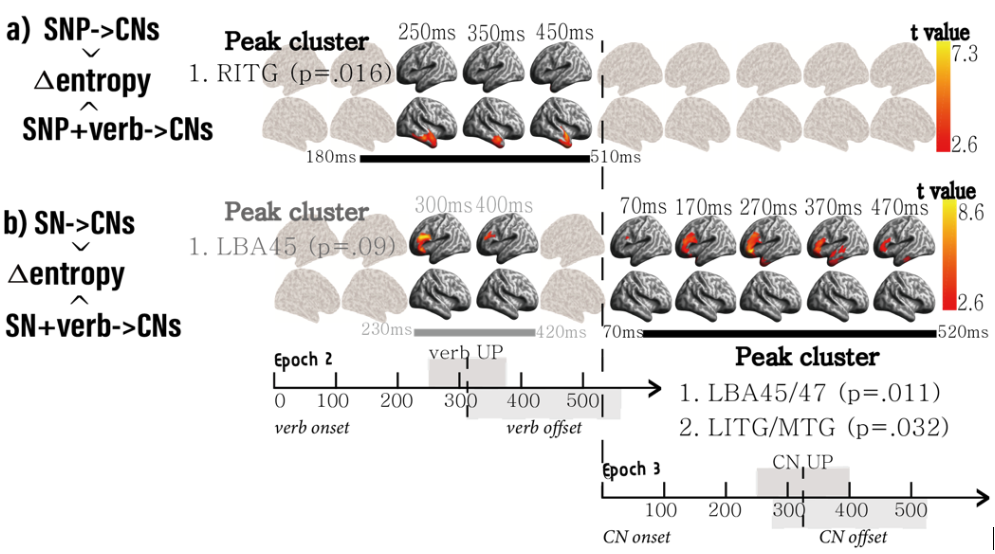


Figure S3-2*: Results of an additional analysis with two different entropy change models. A panel a) at the top shows the clusters found by entropy change of the full-context whereas a panel b) at the bottom shows the entropy change effects captured by the lexical properties of a subject and a verb. The cluster presented in panel b) in grey was not significant after the multiple comparisons correction (p=.09).*


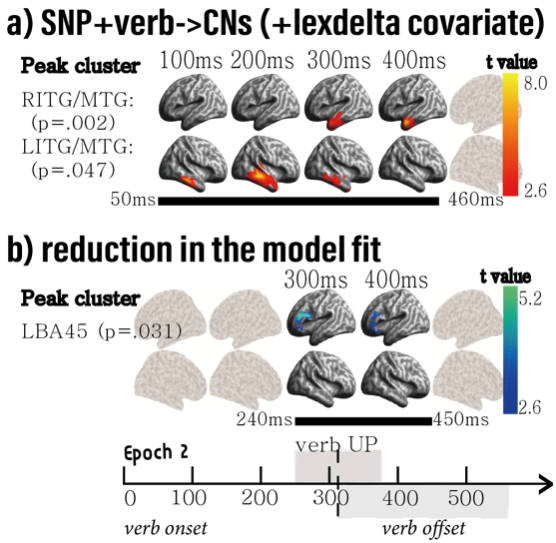


Figure S3-3: *Results of an additional partial correlation analysis by partialling out the covariate of interest when testing the SNP+verb constraint model shown in the panel c) in Figure 5 of the main text. The covariate of interest (denoted as lexdelta in this figure) is the entropy change model based on the lexical properties of a subject (SN) and a verb presented in the panel b) of Figure S3-2). A panel a) shows all clusters found by the SNP+verb constraint in this analysis and a panel b) shows a cluster that was explained away by the covariate.*

**References**

Hagoort P. 2013. MUC (memory, unification, control) and beyond. Front Psychol. 4:416.

Kocagoncu E, Clarke A, Devereux BJ, Tyler LK. 2017. Decoding the cortical dynamics of sound-meaning mapping. J Neurosci. 37:1312–1319.

Lyu B, Choi HS, Marslen-Wilson WD, Clarke A, Randall B, Tyler LK. 2019. Neural dynamics of semantic composition. Proc Natl Acad Sci. 116:21318–21327.
