# Supplementary material for: Decoding the Real-Time Neurobiological Properties of Incremental Semantic Interpretation
Source: Cereb Cortex. 2020 Aug 31;31(1):233–47. doi: 10.1093/cercor/bhaa222 (PMC7727355; doi:10.1093/cercor/bhaa222)
Supplement: CerCor20200002551_SI_section_4_bhaa222 [file cercor20200002551_si_section_4_bhaa222.docx]

**Supplementary Information**

**SI section 4: Cross-covariance decomposition analysis**

The finding that both SNP constraints on verbs and on CNs elicit similar activation in RH temporal regions with similar timing raises the possibility that these constraints are initially activated by the same event representation based on the SNP (see (a) and (b) in Results). If this is the case, we would expect to see some degree of overlap in the predicted semantic space between verbs and CNs; for example, a set of verbs constrained by the preceding SNP would share a similar pattern of topic preferences across stimuli with a set of constrained CNs. To test this hypothesis, we ran an additional cross-covariance decomposition analysis where we projected the blended representations of predicted verbs (see 3.3a in Methods 3.3a) and CNs (see 3.3b in Methods) to a latent subspace that maximally explains the cross-covariance structure between them.

Analysing cross-covariance is one of the simple approaches to quantify the relationship between two multivariate datasets. In this study, we statistically tested if the predicted verbs and CNs based on the SNP context (i.e. blends) share some overlapping semantic space. To do this, we decomposed the cross-covariance between these blends which were centred to have a mean of zero and projected them onto a common subspace that maximally explains the co-variability in the data across topics. However, as our datasets (blends) consist of 50 unique SNPs and 100 topics (features), this approach is highly susceptible to overfitting. To prevent this issue, we carried out 5-fold cross-validation with 100 random partitions splitting the items (trials) into training and test sets for both blends. The training set was used to compute the loading vectors onto which the test set was projected, using singular value decomposition (SVD) of a cross-covariance matrix between the two blends (i.e. The loading vectors were the first left and right singular vectors). Then, we computed a correlation coefficient between the two blends in the projected subspace. Lastly, for statistical testing, we obtained a null-distribution by randomly permuting the items in one of the two blends. This random permutation was repeated 1,000 times and the output correlation coefficient under the null was saved for each iteration.

The result showed a statistically significant correlation between the predicted verbs and CNs once they were projected onto the first canonical basis that maximally explains the cross-covariance (r=0.58; p<.001; see Figure S4). This additional result confirms a largely overlapping semantic space between predicted verbs and CNs, plausibly reflecting the event representation generated by the SNP context.


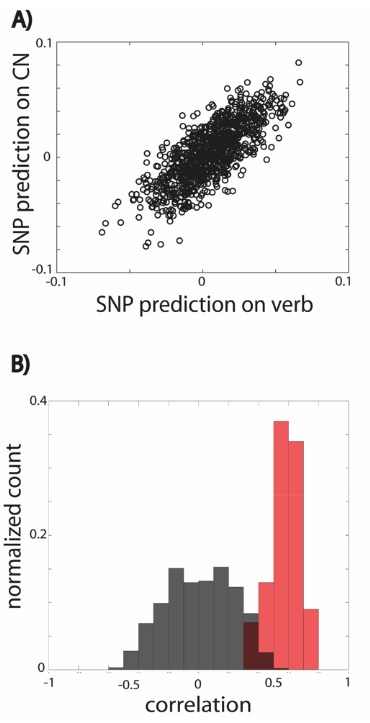


Figure S4: *Panel A) illustrates the relationship between the predicted verbs and CNs (blends) after projecting onto the one-dimensional subspace that maximally explains the cross-covariance between them. The plot concatenated the averaged 10 data-points from the testing set across 100 partitions. Panel B) shows two different histograms: 1) a null-distribution across 1,000 permutations in grey and 2) the actual correlations across 100 partitions in red. We visualised the variability across 100 random partitions to highlight the reliability of our result and the mean correlation value was 0.58.*
