# Supplementary material for: Decoding the Real-Time Neurobiological Properties of Incremental Semantic Interpretation
Source: Cereb Cortex. 2020 Aug 31;31(1):233–47. doi: 10.1093/cercor/bhaa222 (PMC7727355; doi:10.1093/cercor/bhaa222)
Supplement: CerCor20200002551_SI_section_5_bhaa222 [file cercor20200002551_si_section_5_bhaa222.docx]

**Supplementary Information**

**SI section 5: Modelling event-level semantic constraint**

In the main text, we demonstrated how context semantically constrains upcoming words. However, understanding speech not only entails such lexical processing, but it also requires building an event representation depicting the underlying message that the speaker conveys. In order to further investigate the neural underpinnings of early event-level prediction, we constructed a model that captures a joint constraint of the SNP context on verb and CN.

To do this, we blended the topic distributions from the second SNV-CN topic model (see 3.2 in Methods) based on the behavioural responses from the first sentence completion study (see 3.1 in Methods). We first calculated a probabilistic weight $P(verb+CN|SNP)$ by counting each unique continuation response of verb and CN after hearing SNP. We also computed the conditional distributions $P\left( CN topic \right|verb+CN)$ for each unique continuation response, using an element-wise multiplication between the two vectors $P\left( CN topic \right|verb)$ and $P\left( CN topic \right|CN)$. Then, we blended these joint distributions as described in the Methods section 3.3:

$$P\left( CN topic | SNP \right)=\sum_{verb+CN} P\left( CN topic | verb+CN \right)P(verb+CN|SNP)$$

Although this event blend characterizes the same CN topic distribution as the SNP’s non-adjacent constraint on CNs, this particular formulation jointly depicts the semantic constraint of the SNP on verbs and CNs which are optimally expressed in the common latent dimensions (named as $CN topic$ throughout this paper). Then, we calculated the entropy of this event blend as we did for that of SNP constraints on verb and CN separately and tested this model in Epoch 1 and 2 in the exactly same ssRSA analysis pipeline as described in Methods section 4. We obtained the following results (see Figure S5):


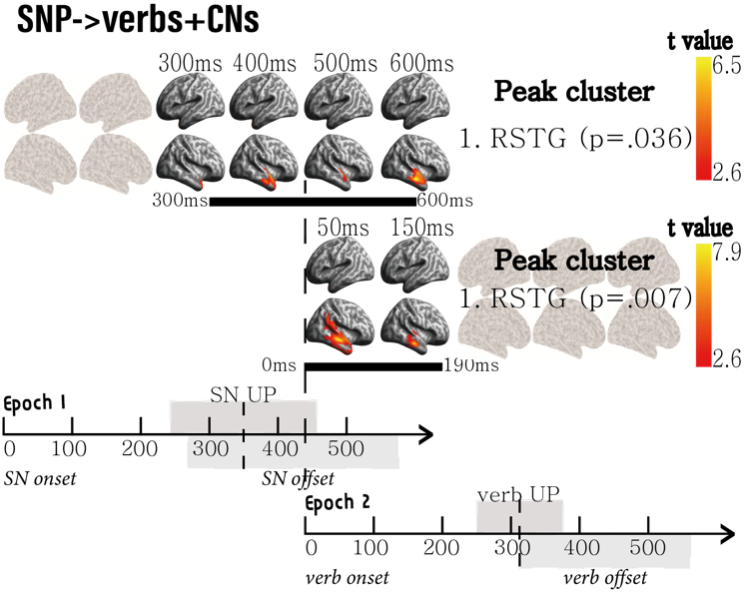


Figure S5: *Results of an additional analysis with the event constraint model (i.e. SNP constraint on the integrated semantics of verbs and CNs). Epoch 1: A significant cluster from 300ms to 600ms after the SN onset, peaking the anterior portion of STG in RH (p=.036). Epoch 2: A significant cluster from 0ms to 190ms after the verb onset peaking at the RH STG (p=.007). See Figure 5 in the main text for more illustrative details of the figure.*

Combining these findings, we can confirm that the overall event-level constraint (i.e. The SNP constraint on combined verbs and CNs) was initially activated in the RH mid-anterior temporal regions approximately as the SN was recognised. This pattern of results is largely consistent with the early SNP constraints on each individual upcoming word including verbs (see panel (a) in Figure 5) and CNs (see panel (b) in Figure 5). However, unlike the SNP constraints on each individual upcoming word, this overall event constraint appears in Epoch 2 in RH mid-STG/MTG areas approximately until 200ms into the verb. This pattern of results supports our interpretation that the early SNP constraint in RH mid-anterior temporal regions not only constrains each individual upcoming word but it also constrains a combination of upcoming words (scenarios). Consistent with our expectation, the event constraint of SNP disappeared before the SNP constraint on CNs represented in RIFG which might reflect maintenance until the verb is integrated into the SNP context.
